# The impact of elective surgical hubs on elective surgery in acute hospital trusts in England

**DOI:** 10.1038/s41467-025-60936-6

**Published:** 2025-07-04

**Authors:** Melissa Co, Tatjana Marks, Freya Tracey, Stefano Conti, Geraldine M. Clarke

**Affiliations:** 1 Improvement Analytics Unit, The Health Foundation, London, England; 2 National Health Service (NHS) England, London, England

**Keywords:** Health policy, Health services

## Abstract

Elective surgical hubs at acute hospital trusts in England aim to increase productivity and efficiency in planned (elective) surgeries, reduce cancellations, and improve patient and staff experiences by ring-fencing care and focussing on High-Volume, Low Complexity (HVLC) surgeries. Using patient-level hospital data from April 2018 to December 2022, we evaluated the impact of new hubs (operational from 2019 onwards) and established hubs (operational before 2019) on trust-wide rates of total and HVLC elective surgeries using a generalised synthetic control methodology. Here, we show that during the first year of operation, the average rate of HVLC elective surgery in trusts with new hubs was 21.9% (95% CI 11.7%, 32.2%) higher than expected. After the COVID-19 pandemic, trusts with established hubs demonstrated greater resilience, with 11.2% higher than expected rates of total (1.3% to 21.2%) and HVLC (1.7% to 20.7%) elective surgery and 0.17 days (0.28 to 0.061) shorter than expected inpatient lengths of hospital stay. Our evaluation provides robust evidence to inform future priorities for elective care delivery.

## Abstract (web summary)

Elective surgical hubs in England aim to improve efficiency, reduce cancellations, and enhance patient and staff experiences by focusing on High-Volume, Low Complexity (HVLC) surgeries. Here, the authors show that new hubs significantly increased HVLC surgery rates and established hubs demonstrated resilience during the Covid-19 pandemic.

## Introduction

As of April 2024, there were 6.33 million people waiting for consultant-led elective (non-urgent or planned) treatments in England^1^. Elective treatments include diagnostic tests, scans, outpatient care, surgery and cancer treatment. A significant reduction in elective care treatments during the COVID-19 pandemic led to record high waiting lists and longer waiting times^2–4^. Delays and cancellations for hospital treatment can negatively impact patients’ physical and mental health^5^, increase the risk of deterioration, and reduce treatment options^6^. Key priorities for National Health Service (NHS) England in 2023/24 were to reduce waiting times and deliver 30% more elective treatments than pre-pandemic levels by 2024/25^7^.

Elective surgical hubs (hereafter called hubs) play a pivotal role in NHS England’s plans to tackle the COVID-19 backlog of care and reduce waiting times^8^. Hubs are specialised centres within hospitals dedicated to elective surgeries, with staff and resources protected from being used for emergency surgeries (a practice known as ‘ringfencing’). They can be located within a hospital building in a separate area from emergency surgery, in a separate building, or on a separate site away from the main hospital. Clinicians in the NHS have advocated ring-fencing elective from emergency surgery to improve productivity and ease pressure on the acute sector since the early 1990s^9^. Similar ringfencing approaches have been used across the UK and other countries including Ireland, Norway, Canada and Australia^10–15^.

In 2020, amid and after the COVID-19 pandemic, many hubs were opened in England to avoid disruption of elective care and enhance infection control^16^. In May 2021, Getting It Right First Time (GIRFT), a national NHS programme designed to improve treatment and care of patients, introduced the High-Volume Low-Complexity (HVLC) programme to coordinate new and existing hubs at a national level and provide a framework for delivery^17^. Initially, the focus of hubs in the programme was to perform HVLC elective surgeries across six specialties: ophthalmology, general surgery, trauma and orthopaedics (including spinal surgery), gynaecology, ear, nose and throat, and urology. Although the scope has since broadened to cover other specialties and more complex patients, hubs are still required to deliver at least one HVLC specialty^18^. In 2022, GIRFT introduced the Elective Hub Accreditation Programme to provide a formal assessment of existing hubs against a core set of clinical and operational standards. Capital injection to drive the expansion of the hub model has included funding from the Targeted Investment Fund (TIF), a £1.5B investment toward recovering elective care services between 2023 and 2025^18^. As of September 2024, there were 108 hubs operating in NHS hospital trusts (organisational units within the NHS) across England, with a further 29 planned to open by the end of 2025. The distribution of hubs across the country differs widely by configuration, capacity, care provision and contextual setting within acute hospital trusts^16^.

The decision to set up an elective surgical hub can depend on several factors. These include funding and resources. Trusts that bid for TIF funding were required to develop detailed proposals outlining needs, benefits and potential outcomes as well as comprehensive financial plans. Better-financed trusts, or those successful in bidding for funds, may be more likely to set up a hub, or be running additional initiatives targeting efficiency improvements. Additionally, trusts with higher patient demand or waiting lists, or those with a history of successful elective surgery programmes, may prioritise the creation of a hub. Finally, different trusts may have different strategic priorities, management structures and operational capacity all of which may play a role in the decision to set up a hub and other initiatives. Against this background, COVID-19 also acted as a catalyst for trusts to open a hub—creating clearly demarcated areas: one for acute activity, including COVID-19 patients, and another fully dedicated to elective activity with ringfenced staff and operating theatres.

**Fig. 1:**
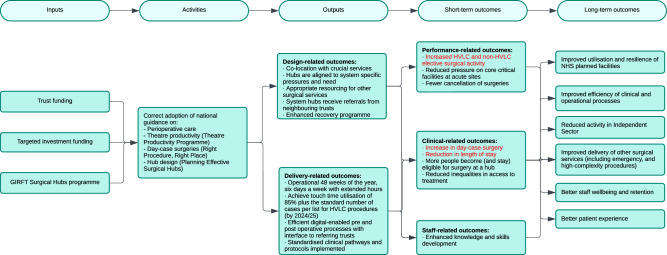
Logic model for elective surgical hubs national programme. Theory of change outlining the resources and activities (inputs) required to establish and maintain the hubs, the services and interventions provided (outputs) and the potential short-term and long-term impacts on patient care and the system efficiency (outcomes). Short-term outcomes in red are those that we focused on in this evaluation. Source data are provided as a Source Data file.

The GIRFT HVLC programme aims to enable hubs to achieve several projected short-term outcomes, including performance-related improvements such as increased activity and fewer cancellations; clinical benefits, such as more day-case surgeries, shorter post-surgery stays and reduced inequalities in access to treatment; and staff-focused benefits such as enhanced skills and knowledge development, predictable working patterns, and better wellbeing and retention (Fig. 1)^19^. Ultimately these changes are expected to result in better longer-term utilisation and resilience of NHS planned facilities and resources, along with greater efficiency.

Evidence on the impact of hubs, or their international equivalents, on potential outcomes is heterogenous and limited. A rapid review of 12 national and international studies published between 2015 and 2022 found evidence to suggest that hubs can improve clinical outcomes such as length of hospital stay, operative and post-operative complications, readmission rates and surgery quality, and found limited evidence of hubs on performance outcomes such as efficiency, utilisation, volume, cancellations, and time from diagnosis to treatment^20^. Other studies have reported reduced length of stay after surgery^11–13,15,21,22^. A study of three hospitals in Norway suggested ringfencing can improve hospital efficiency^14^. A study in Canada found that continuity of care and patient satisfaction could be maintained when introducing additional ring-fenced capacity at a single site^10^. However, most studies were focused on a single location with considerable variation in the types of hubs and surgical specialties analysed, leading to a lack of generalisability. Additionally, they often used weak methods like before-and-after designs, which can lead to incorrect conclusions about effects. Only one study attempted to examine the causal impact of hubs^14^.

The increasing number of hubs and investment in new sites warrants robust causal evidence of their impact on key outcomes. The national rollout provides the opportunity to assess causal impacts across the wider health care system by comparing trusts with hubs to those without. Here, we perform a causal evaluation of the impact of hubs on trust-wide elective care performance (elective surgery rate) and clinical (inpatient length of hospital stay and day-case proportion) outcomes nationally using the generalised synthetic control method (GSC)^23^. The GSC approach is suitable for evaluating the impact of a health care intervention (in this case having a hub) against a counterfactual (if the hub did not exist) where there are multiple treated units, staggered intervention start dates, and data is available at multiple time points. This modelling framework effectively accounts for unique aspects of typical health care data, namely, the likely presence of unmeasured confounders and of effects that vary across time and trusts^24,25^.

In our analysis, we distinguish between ‘established’ hubs that opened long before the COVID-19 pandemic (operational before 2019) and ‘new’ hubs that opened just before, or in response to, the pandemic (operational from 2019 onwards). Trusts with established hubs (hereafter referred to as established-hub trusts) likely had set procedures in place before the start of the pandemic in March 2020, potentially positioning them better than other trusts to recover elective surgeries lost during the government-mandated COVID-19 lockdown. In contrast, trusts with new hubs (hereafter referred to as new-hub trusts), many of which opened during lockdown, typically in response to the suspension of planned services during the lockdown, may have taken time to establish their operational standards after the lockdown. We examined impacts on all (or total) elective surgery and, assuming that hubs focus on HVLC elective procedures, we examined HVLC elective surgery separately to better understand the specific contribution of hubs to the overall effect observed across each trust.

## Results

A total of 8,077,669 elective surgeries at 31 new-hub trusts, 23 established-hub trusts and 54 trusts without a hub (non-hub trusts) between April 2018 and December 2022 (excluding lockdown months April 2020 to March 2021) were included in our study (Supplementary Figs. 1 and 2). An earlier mapping study of hubs in England found that they are widespread across NHS regions in England, mainly in more densely populated areas, with significant variation in the hub types, specialties, care provision, and their healthcare provider contexts^16^. Characteristics of trust catchment populations for trusts with and without hubs were broadly similar. However, trusts without hubs (non-hub trusts) tended to have smaller median catchment population size (279,429 vs 400,106 and 426,938) and larger median proportion of individuals with White ethnicity (93% vs 85% and 89%) than new-hub and established-hub trusts, respectively (Table 1).

**Table 1:** Comparison of population characteristics in new-hub, established-hub and non-hub trusts in England in 2019

	New-hub trusts (*N* = 31) Median (IQR)	Established-hub trusts (*N* = 23) Median (IQR)	Non-hub trusts (*N* = 54) Median (IQR)
Population from a White ethnic background (%)	85 (72, 92)	89 (79, 93)	93 (87, 96)
Population who are male (%)	48 (48, 50)	50 (48, 51)	48 (48, 50)
Population who are aged 65+ years old (%)	19 (14, 21)	19 (16, 21)	20 (18, 23)
Catchment population	400,106 (295,955, 579,440)	426,938 (319,217, 612,212)	279,429 (195,782, 462,603)

**Fig. 2:**
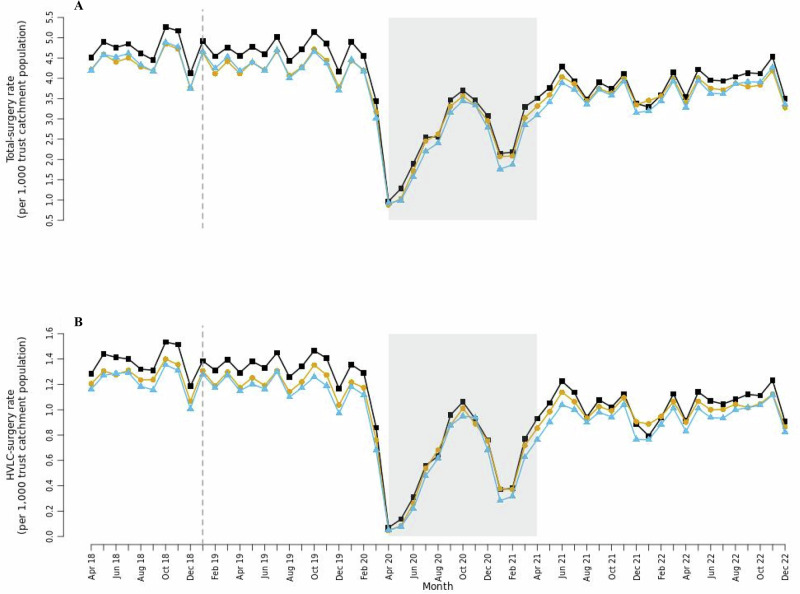
Trends in elective surgery rates over time. Trends in (**A**) total elective surgery and (**B**) HVLC elective surgery rates in 31 new-hub, 23 established-hub and 54 non-hub trusts between April 2018 and December 2022 in England. Grey shaded area indicates timing of the UK government COVID-19 lockdown period between April 2020 and March 2021. Dashed vertical lines indicate the time point for the opening of new hub trusts. Source data are provided as a Source Data file.

The rate of total elective surgery and HVLC elective surgery (hereafter referred to as total-surgery and HVLC-surgery respectively) were consistently higher in non-hub trusts than in new-hub and established-hub trusts throughout our study period (Fig. 2). In the two years leading up to the pandemic, the average total-surgery rate at non-hub trusts (4.67 surgeries per 1000 trust catchment population per month [SD = 1.36]) was 8% higher than in new-hub trusts (4.31 [SD = 1.14]) and 9% higher than in established-hub trusts (4.3 [SD = 1.25]) (Table 2). Post-lockdown, the average total-surgery and HVLC-surgery rates decreased across all trust types, but larger decreases were observed in non-hub (−17.3% for total and −22.1% for HVLC) than in both new-hub (−15.8% and −20.3%) and established-hub trusts (−13.8% and −18.3%), making rates more similar across all types of trust post-pandemic compared with pre-pandemic. Excluding the COVID-19 lockdown period, approximately 65−75% of all surgeries were treated as a day-case with established-hub trusts consistently having the lowest average across all surgeries but no tangible differences between trust types for HVLC surgeries. (Supplementary Fig. 3). Pre-pandemic, non-hub trusts had the shortest average inpatient length of hospital stay (hereafter referred to as length of stay) for all surgeries, including HVLC (Supplementary Fig. 4). Post-pandemic, new-hub and established-hub trusts showed the greatest reductions in length of stay.

**Table 2:** Comparison of elective activity in trusts in England before (April 2018 to March 2020) and after (April 2021 to December 2022) the UK government COVID-19 lockdowns between April 2020 and March 2021

	New-hub trusts			Established-hub trusts				Non-hub trusts	
	April 2018 - March 2020	April 2021 -December 2022	Difference	April 2018 - March 2020	April 2021 - December 2022	Difference	April 2018 - March 2020	April 2021 - December 2022	Difference
	Mean (SD)	Mean (SD)	**%**	Mean (SD)	Mean (SD)	**%**	Mean (SD)	Mean (SD)	***%***
**Total elective activity**									
Elective surgical rate (per 1000 trust catchment population per month)	4.31 (1.14)	3.63 (0.91)	−15.8%	4.3 (1.25)	3.7 (1.2)	−13.8%	4.67 (1.36)	3.86 (1.3)	−17.3%
Day-case proportion	0.77 (0.05)	0.77 (0.07)	0.0%	0.74 (0.07)	0.74 (0.08)	0.0%	0.76 (0.07)	0.77 (0.07)	1.2%
Inpatient length of stay (mean days)	3.33 (0.66)	3.27 (0.7)	−1.9%	3.4 (0.69)	3.33 (0.77)	−2.0%	3.18 (0.65)	3.19 (0.77)	0.3%
**HVLC elective activity**									
Elective surgical rate (per 1000 trust catchment population per month)	1.18 (0.4)	0.94 (0.35)	−20.3%	1.22 (0.52)	1 (0.52)	−18.3%	1.34 (0.49)	1.04 (0.46)	−22.1%
Day-case proportion	0.7 (0.07)	0.69 (0.09)	−0.4%	0.69 (0.08)	0.69 (0.09)	−1.3%	0.69 (0.1)	0.7 (0.11)	2.5%
Inpatient length of stay (mean days)	2.62 (0.46)	2.39 (0.49)	−8.5%	2.67 (0.56)	2.42 (0.52)	−9.5%	2.61 (0.47)	2.47 (0.58)	−5.3%

### New-hub trusts

Applying the generalised synthetic control model, estimated effect sizes for the impact of opening a new hub on total-surgery and HVLC-surgery rates during the first 12 months were similar (each estimated an additional 0.17−0.18 surgeries per 1000 trust catchment population per month), although  these were only significant for HVLC-surgery (Table 3). The HVLC-surgery rate was 0.169 surgeries per 1000 trust catchment population per month (95% confidence interval = 0.090 to 0.248) higher in new-hub trusts during the first 12 months compared with the synthetic control. This is equivalent to a 21.9% (95% CI = 11.7% to 32.2%) higher than expected increase in HVLC-surgery in new-hub trusts when compared with a synthetic control with similar pre-opening trends.

**Table 3:** Estimated average effect of elective hubs on elective activity in new-hub trusts for 12 months post-opening between January 2019 and December 2022 in England

New-hub trusts	Synthetic control	Hub-trusts	Coefficient	95% CI	Change	95% CI	*p*-value
**Total elective activity**							
Total-surgery rate (per 1000 trust catchment population per month)	3.422	3.598	0.176	−0.115 to 0.467	5.1%	−3.4% to 13.7%	0.236
Day-case proportion	0.748	0.768	0.020	0.002 to 0.038	2.7%	0.3% to 5.1%	0.027*
Inpatient length of stay (days)	3.340	3.312	−0.029	−0.151 to 0.094	−0.9%	−4.5% to 2.8%	0.646
**HVLC activity**							
HVLC-surgery rate (per 1000 trust catchment population per month)	0.772	0.942	0.169	0.090 to 0.248	21.9%	11.7% to 32.2%	<0.001***
Day-case proportion	0.686	0.696	0.010	−0.012 to 0.032	1.5%	−1.8% to 4.7%	0.383
Inpatient length of stay (days)	2.399	2.399	<0.001	−0.128 to 0.128	0.0%	−5.3% to 5.3%	0.997

Coefficients are the estimated average effect of opening a hub during the first 12 months of opening for hubs opening between January 2019 and December 2022 excluding the UK government COVID-19 lockdown period between April 2020 and March 2021. Estimates are derived from the generalised synthetic control model. 95% confidence intervals and p-values are derived from non-parametric bootstrap pairwise difference two-sided T tests, unadjusted for multiple comparisons. Effect estimates are statistically significant at * *p* < 0 .05, *** *p* < 0.001.

**Fig. 3:**
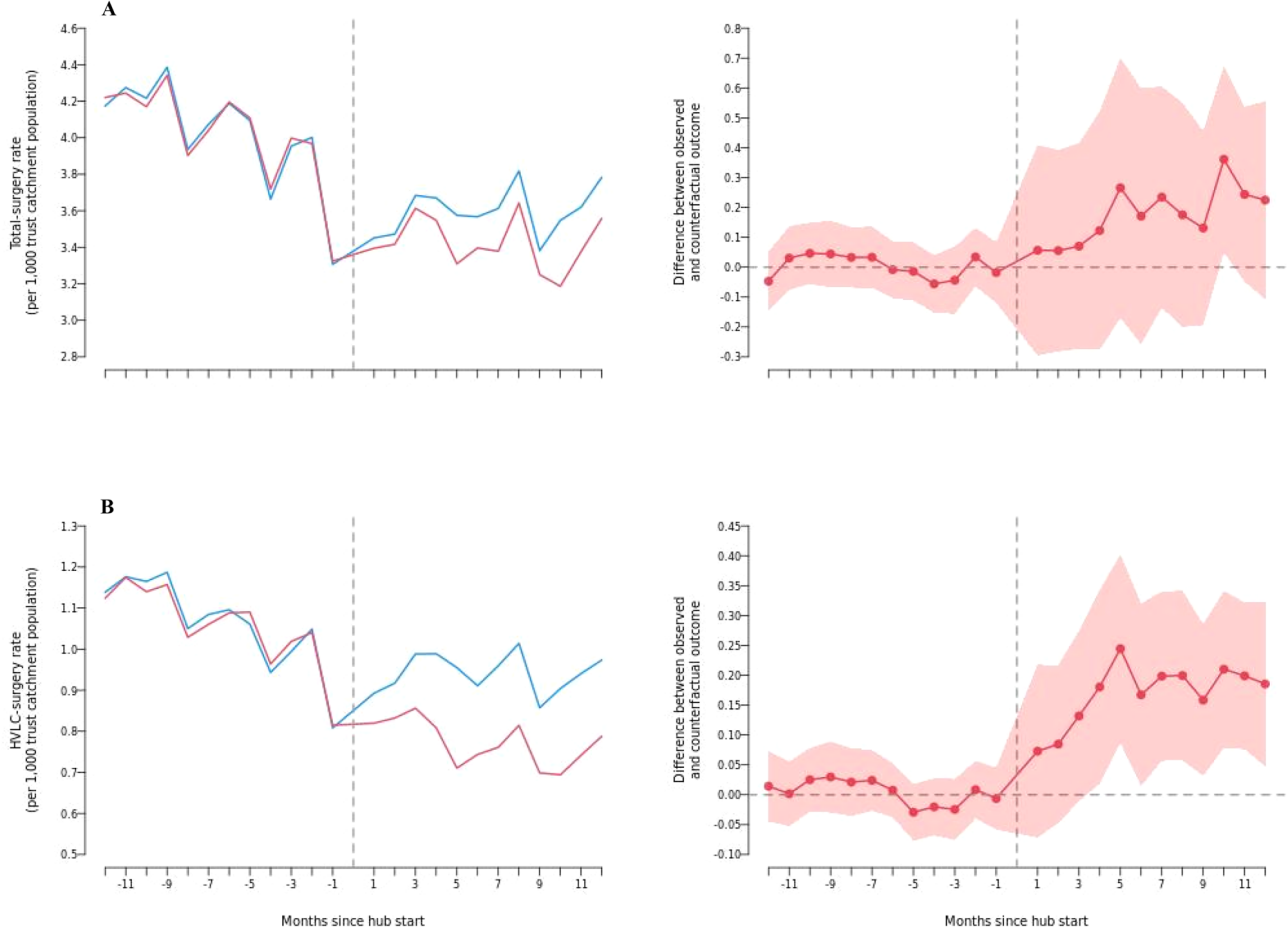
Estimated effects in new-hub trusts. Estimated effect of elective hubs on (**A**) total elective surgery and (**B**) HVLC elective surgery rates in 31 new-hub trusts during 12 months pre- and post-opening for hubs opening from January 2019 onwards in England. Results exclude the UK government COVID-19 lockdown period between April 2020 and March 2021. Estimates are derived from the generalised synthetic control model. Dashed vertical lines indicate the time point for the opening of new hub trusts. Shaded areas show non-parametric bootstrap 95% confidence intervals around effect estimates. Source data are provided as a Source Data file.

Figure 3 displays observed and counterfactual trends for total-surgery and HVLC-surgery rates (left panels), as well as corresponding impact estimates (right panels), at new-hub trusts. Throughout the pre-intervention period, rates of both total-surgery and HVLC-surgery matched the synthetic control, indicating a good model fit. However, these rates steadily declined, reflecting that the pre-intervention months for many new hubs were just before lockdown, when surgeries were paused (Fig. 2). Positive effects on HVLC-surgery rates were only significant after three months and were driven by a continued reduction in the synthetic control, compared to a marked increase at new-hub trusts.

For all surgeries, the day-case proportion was an average of 0.020 (95% CI = 0.002 to 0.038) higher in new-hub trusts compared to the synthetic control during the first 12 months of opening. There was no significant effect on the day-case proportion for HVLC-surgery or on length of stay for either total-surgery or HVLC-surgery in new-hub trusts during this period (Supplementary Figs. 5, 6).

### Established-hub trusts

For established-hub trusts, the total-surgery rate was 0.374 (95% CI = 0.043 to 0.705) surgeries per 1000 trust catchment population per month higher between April 2021 and December 2022 compared with the synthetic control (Table 4). This corresponds to an average increase of 11.2% (95% CI = 1.3% to 21.2%) in all surgeries post-lockdown, due to having an established hub. Similar trends were also seen for the HVLC-surgery rate with an average increase of 11.2% (95% CI = 1.7% to 20.7%) during the post-lockdown period compared with the synthetic control.

**Table 4:** Estimated average effect of elective hubs on elective activity in established-hub trusts between April 2020 and December 2022 in England

Established-hub trusts	Synthetic control	Hub-trusts	Coefficient	95% CI	Change	95% CI	*P*-value
**Total activity**							
Total-surgery rate (per 1000 trust catchment population per month)	3.330	3.704	0.374	0.043 to 0.705	11.2%	1.3% to 21.2%	0.027*
Day-case proportion	0.725	0.743	0.018	−0.007 to 0.043	2.5%	−0.9% to 5.9%	0.149
Inpatient length of stay (days)	3.453	3.330	−0.124	−0.259 to 0.012	−3.6%	−7.5% to 0.3%	0.074
**HVLC activity**							
HVLC-surgery rate (per 1000 trust catchment population per month)	0.897	0.998	0.101	0.016 to 0.186	11.2%	1.7% to 20.7%	0.020*
Day-case proportion	0.674	0.685	0.011	−0.014 to 0.036	1.6%	−2.1% to 5.3%	0.385
Inpatient length of stay (days)	2.585	2.415	−0.170	−0.280 to −0.061	−6.6%	−10.8% to −2.3%	0.002**

Coefficients are the estimated effects of an established hub on planned surgeries after the end of the UK government COVID-19 lockdown in April 2021 until December 2022. Estimates are derived from the generalised synthetic control model. 95% confidence intervals and p-values are derived from non-parametric bootstrap pairwise difference two-sided T tests, unadjusted for multiple comparisons. Effect estimates are statistically significant at * *p* < 0.05, ** *p* < 0.01.

**Fig. 4:**
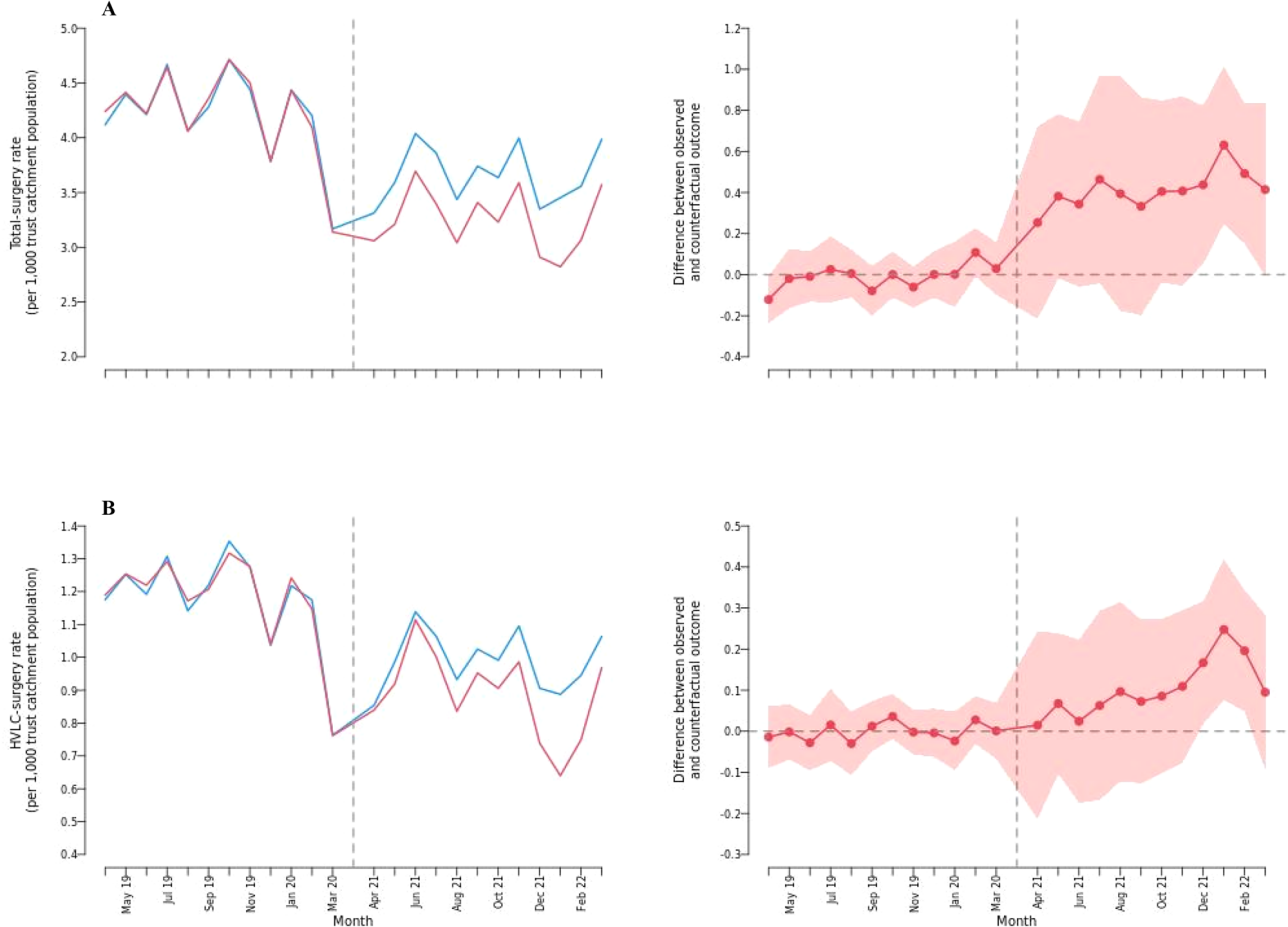
Estimated effects in established-hub trusts. Estimated effect of elective hubs on (**A**) total elective surgery and (**B**) HVLC elective surgery rates in 23 established-hub trusts between April 2018 and December 2022 in England. Results exclude the UK government COVID-19 lockdown period between April 2020 and March 2021. Estimates are derived from the generalised synthetic control model. Dashed vertical lines indicate the time point for the start of the analysis. Shaded areas show non-parametric bootstrap 95% confidence intervals around effect estimates. Source data are provided as a Source Data file.

As for new-hub trusts, both total-surgery and HVLC-surgery rates matched the synthetic control during the pre-intervention period indicating a good model fit (Fig. 4). Post-intervention, the positive effects on total-surgery and HVLC-surgery rates steadily increased, driven by a faster initial increase in rates in the established-hub trusts not matched over the study period in the estimated synthetic control.

The average length of stay for HVLC-surgery was 0.170 days (95% CI = −0.280 to −0.061) shorter in established-hub trusts than in the synthetic control. There were no significant effects on length of stay across all surgery types at established-hub trusts, nor on the proportion of day cases for either total-surgery or HVLC-surgery (Supplementary Figs. 7, 8).

### Specialties and individual trusts

When examining each HVLC specialty in turn, we only found a significant effect on the rate of general surgery at new-hub trusts and no significant effect on any individual specialties at established-hub trusts. General surgery comprises 13% of all HVLC elective surgery in new-hub trusts and increased by 0.023 surgeries per 1000 trust catchment population per month (95% CI = 0.007 to 0.039) during the first 12 months of opening compared with the synthetic control (Supplementary Table 4). When looking at individual trusts, we were unable to detect significant impacts of opening a new hub or having an established hub on elective activity at most trusts (Supplementary Figs. 9, 10).

### Sensitivity analyses

Results from the robustness and sensitivity tests are including in the Supplementary Material (Supplementary Table 2). We found no significant effects in placebo tests, and results remained largely consistent when changing how we accounted for lockdown or when adjusting for different specialty mixes.

## Discussion

This evaluation examines the national impact of hubs on elective surgical activity in trusts across England. Analysis conducted at trust level allowed us to assess impacts on both the trust and the overall system. Improvements in elective activity were observed in new-hub trusts during their first 12 months of opening (excluding lockdown) and in established-hub trusts post-lockdown, using robust causal statistical methods. These results can be used to inform policy and service planning for elective care delivery.

HVLC elective surgery was 21.9% higher at 31 new-hub trusts during the first year of opening a hub (excluding lockdown) than in the estimated synthetic control. Nationally, this amounts to 29,182 more HLVC procedures per year (95% CI = 15,449 to 42,915) than expected at trusts serving approximately 23% of England’s population (14 million people). The effects took about three months to materialise, likely because new hubs needed time to become fully utilised and effective. This is consistent with the common practice of starting with a smaller patient cohort when opening a new hospital unit to ensure all processes are functional, and staff acclimate to the new environment and workflows. Since hubs initially focused on HVLC procedures, increases in HVLC elective surgical rates are likely to reflect effects directly attributable to the hub. Opening a new hub didn’t significantly affect the overall rate of elective surgeries. However, the estimated increase in the rate of total and HVLC elective surgeries at new-hub trusts compared to the synthetic control was similar, suggesting, firstly, that any rise in total surgeries was mainly due to more HVLC surgeries and, secondly, that there was no negative impact of opening a hub on non-HVLC elective surgeries in the wider trust.

At 23 established-hub trusts, both total and HVLC elective surgery rates were 11.2% higher following the lockdown than in the estimated synthetic control. This amounts to 51,086 more elective surgeries per year (95% CI = 5523 to 98,030) than expected at trusts serving around 17% of England’s population (11.5 million people). Trusts with established hubs may have recovered elective surgery faster after lockdown because they already had embedded processes, structures, and staffing in place before the pandemic. This may have helped them perform more surgeries and discharge patients earlier than they otherwise could have. Increases in both total and HVLC elective surgery rates could be explained by the focus of hubs shifting over time to non-HVLC elective surgeries or by other effects indirectly attributable to hubs. For example, as hubs became more established, and procedures were streamlined, trusts may have begun focusing on improving efficiency in activity in other areas.

As well as higher rates, we also found evidence of shorter inpatient stays at established-hub trusts suggesting that hubs also have the potential to improve overall efficiency in elective care delivery. The private sector is used to increase NHS treatment capacity and reduce the waiting list. Typically, it is the procedures which are easier to deliver at scale and fit well within hubs which are outsourced to the private sector, leaving the more complex patients for the NHS^26^. This practice is sometimes referred to as ‘cherry-picking’. With increased activity and efficiency, hubs may offer the potential to reduce the NHS reliance on the independent sector and repatriate some activity. However, effects on length of stay outcomes were not consistent across both all surgery types at either new-hub or established-hub trusts. Reducing length of stay is a key focus of NHS England^27^ and the mixed significance of effects may reflect the influence of other concurrent initiatives aimed at reducing length of stay in both hub and non-hub trusts.

When looking at individual HVLC specialties, we only found statistically significant effects of the impact of opening a new hub on general surgery. Most other specialty-specific analyses likely had samples too small to detect a difference. We were also unable to discern any reliable impacts at individual trusts. This may be because the data we had did not allow us to clearly identify hub-specific activity, or to account for some of the differences between hubs that change over time or other interventions occurring elsewhere within a trust.

Our evidence is consistent with previous studies showing ring fencing or surgical hub equivalents can increase surgery volumes^28^ and reduce inpatient length of hospital stay^11–13,15,21,22^. Conversely, Vanhegan et al. reported a decreased rate of surgeries per surgeon list in a surgical hub equivalent set up to relieve pressure at an acute site in England^29^. However, results from these studies are typically not generalisable due to a focus on a single site or specialty, or the use of weak methods such as before-and-after designs. Moreover, the national healthcare system, local contexts, and even hospital-specific contexts of these studies differs greatly, and it is difficult to extrapolate their findings to English hospital settings.

The generalised synthetic control method offers advantages over other causal inference techniques. Most importantly, this methodology does not rely on the parallel trends assumption and can control for time-varying effects, including national changes in policy or regional variation in trends over the study period reducing potential biases in our estimates^23^. Further, by allowing for analysis of multiple trusts simultaneously, we were able to detect effects not apparent in individual trusts. Finally, by deriving the synthetic control empirically, we minimise the risk of researcher bias that might arise from manually selecting non-hub trusts as controls.

Our evaluation has some limitations. Firstly, we were unable to distinguish between elective activity taking place at a hub and the wider trust. To investigate this, we estimated effects on HVLC procedures, which were assumed to be the main focus of hub activity. Secondly, we could not account for movement of activity to the private sector, transfers of activity between trusts, operational changes in trusts, or other changes to the trust catchment area, which may influence trust activity. These issues could lead to residual unobserved confounding that could bias our estimates. Thirdly, we were unable to account for systemic differences between trusts, such as their decision to set up a hub or other broader investments in elective care that may go alongside that. These systemic differences could confound the relationship between hubs and improved outcomes. For example, broader investments in elective care may influence whether trusts open a hub and may also separately be associated with increased elective surgical activity outside the hub. There may also be selection bias if the hub trusts are not representative of trusts without hubs. Fundamental differences in setup and funding between hub trusts and non-hub trusts may mean that the effects found in our study may not be generalisable to non-hub trusts. Similarly, we could not account for other interventions or local initiatives related to elective care in non-hub trusts, which could dilute the effects we estimated. The generalised synthetic control method attempts to control for unmeasured time-varying confounders and may reduce these biases but may not eliminate them. Fourthly, in the new-hub trusts analysis, we could not determine whether increased rates reflected greater efficiency from setting up a hub or simply increased capacity from additional resources. However, the improvement in the day-case proportion across all elective surgeries at new-hub trusts might indicate the former. Finally, the COVID-19 pandemic severely disrupted the whole health system. Elective care was paused, and elective activity still had not recovered at the time of our evaluation. Many hubs opened during this time to aid with infection control as well as to reduce the disruption to services. In our analyses, we accounted for the immediate effects of pausing elective care by removing the government lockdown period and performing sensitivity analyses to understand how modelling different time periods might affect the results. Although our models aim to account for these disruptive trends, we cannot discount the effect they may have had on our results and their generalisability to future contexts.

Further research is required to understand other impacts of elective surgical hubs (Fig. 1), such as the effects on staff-related outcomes and inequalities in access to treatment, as well as any unintended consequences. This includes examining the quality and safety of care for patients in hubs, such as whether readmission or mortality rates have changed, as well as understanding if there are unintended consequences on outcomes for patients treated outside of hubs, such as those with higher-complexity surgeries not offered in hubs. Understanding how resources may have shifted and if this has affected efficiency, safety, and patient outcomes in the wider system is important. Since hubs were designed to protect planned care resources from the overflow of emergency care, it will be important to examine whether improvements in elective surgery rates have led to any adverse effects on emergency care as well. Evaluations with longer follow-up time (particularly after the lockdown) will help to disentangle the effects of the pandemic disruptions to elective care and the longer-term impacts of hubs. Future research could examine whether hubs are achieving other aims laid out by GIRFT, including reducing pressure at acute sites and improving staff wellbeing and training opportunities^18^. Qualitative research on what factors make hubs successful will also be important in identifying best practices.

## Methods

Ethical approval by an Institutional Review Board, written informed consent or participant compensation was not required for this study as it utilised secondary data that had been previously collected and anonymised. No new data collection was conducted, and all data used were in compliance with relevant ethical guidelines.

### Study design

We evaluated the impact of hubs on trust-wide elective activity. Our unit of analysis was a trust. Treated units comprised trusts with hubs (hub-trusts); control units comprised trusts without hubs (non-hub trusts). Our outcomes were the elective surgical rate (defined as the number of elective surgeries per 1000 trust catchment population), the inpatient length of hospital stay (defined as the length of stay in days following admission for overnight stay), and the day-case proportion (defined as the proportion of elective surgeries without overnight stay (Fig. 1). We examined each outcome for all elective surgeries taking place across a trust (referred to as total elective surgery), for the subset of HVLC elective surgeries only, and for each specialty in turn (Supplementary Table 1). For total elective surgery, the aim was to understand the trust-wide impact of the introduction of hubs. This takes account of any spillover effects, where the introduction of a hub might have affected the delivery of elective surgery elsewhere in the trust. We used HVLC elective surgery, which was the initial focus of hubs, as a proxy for hub activity and the aim here was to delineate the trust-wide effects attributable to the hubs.

We distinguish between trusts with established hubs (opened before 2019), with new hubs (opened 2019 onwards) and without hubs.

- For established-hub trusts, we examined impacts from April 2021 onwards, corresponding with the relaxation of lockdown restrictions imposed by the UK government. Hence the pre-intervention period spans a fixed 24 months from April 2019 to March 2020 and the post-intervention period spans a fixed 21 months from April 2021 to December 2022.
- For new-hub trusts, we examined impacts during the first 12 months of opening, using event time rather than calendar time to account for staggered opening. The pre-intervention period ranges between 11 months (from April 2018 to March 2019 for the first new hub which opened in March 2019) and 40 months (from April 2018 to August 2022 excluding April 2020 to March 2021 for the last new hub which opened in August 2022). The post-intervention period ranges from 12 months for hubs opening before December 2022 and 5 months for the last hub opening in August 2022.

### Data sources

We used patient-level episode data for the NHS in England from the Hospital Episode Statistics (HES) admitted patient care dataset. HES is the main source of data on admissions at NHS Hospitals in England. It includes detailed records of all inpatient admissions across NHS hospitals in England. We included all episodes for patients aged 17 years or over between April 2018 and December 2022 to allow for both pre- and post-intervention data. Each episode represents the time spent by one patient at a single hospital under a single consultant. We linked all episodes within the same hospital admission to generate spells. We included spells with an episode assigned an Office of Population Censuses and Surveys version 4 (OPCS4) code representing an ‘intermediate’ surgery, as defined by Abbott et al.^30^. This includes procedures routinely undertaken in an operating theatre and/or under anaesthesia. We used information provided by GIRFT to identify HVLC activity (Supplementary Table 1). Patient-level spell data was aggregated to trust level to create monthly data series.

GIRFT provided information on hub opening dates and specialties performed. Demographic characteristics of the trust catchment population including the proportions aged 65 years and older, male and with White ethnicity were obtained from the Office for Health Improvement and Disparities’ NHS Acute (Hospital) Trust Catchment Populations dashboard^31^. These were available yearly for 2018, 2019 and 2020; 2020 demographic information was applied to 2021 and 2022. Characteristics of patients undergoing elective surgery (age, gender, deprivation and number of Elixhauser comorbidities over the preceding 24 months) were obtained for each trust for each month from HES.

We excluded trusts with data missing in one or more months, or those with known organisation changes likely to affect data (e.g., trust mergers). We also excluded trusts specialising in a single surgical area due to less generalisable activity (Supplementary Fig. 1).

### Statistical analysis

We used the generalised synthetic control method (GSC) to estimate the impact of new and established hubs on trust-wide elective activity^23^. We chose the GSC modelling framework based on the attributes of our data and findings from recent simulation studies that have compared the relative performance of difference-in-difference, GSC and other causal estimation approaches for this type of data^24,32^. GSC offers flexibility for causal inference in our context for the following reasons:

- It accounts for unique aspects of typical health care data, namely, the likely presence of unmeasured confounders and of effects that vary across time and trusts. For example, in our data this might include differences between trusts in relation to why they choose to open a hub or not, or other time-varying differences between trusts that could affect outcomes.
- It allows for multiple treated units, enhancing the power to detect effects and allowing for mitigating the impact of any potential idiosyncrasies that a few hub trusts may show compared to others.
- It supports varying treatment periods among treated units, which is useful for incorporating staggered hub opening dates in the new-hub trusts analysis.
- It conveniently incorporates observed time-varying covariates by including them as main effects in the model.
- It provides coherent measures of accuracy, such as standard errors and confidence intervals for effect estimates, using a parametric bootstrap procedure.

GSC uses interactive fixed effect regression modelling and synthetic control methodology to estimate the outcome changes in hub-trusts relative to a synthetic control, or counterfactual, which estimates the outcomes trends that would have been seen in the trust without the hub. Synthetic controls are constructed using data from non-hub trusts across all time points and data from hub-trusts in the pre-intervention period only. Estimates are aggregated across hub-trusts to obtain pooled system estimates of effect. Confidence intervals around estimated effects are derived using a parametric bootstrap approach (1000 runs). Technical details of the GSC method are described extensively in the seminal work by Xu^23^ and associated tutorial (https://yiqingxu.org/packages/gsynth/articles/tutorial.html) as well as in simulation and evaluation studies^24,25,32,33^. We also provide more information for the interested reader in Appendix 3 of the Supplementary Material.

Standard diagnostic checks were performed to test the validity of modelling assumptions^34^. Effect estimates were adjusted to control for changes over time in the population-at-risk characteristics. For elective activity rates, the population-at-risk is the trust catchment population, and we adjusted for the proportion of those who were male, with White ethnicity, and aged 65 years or older. For day-case proportion and inpatient length of stay, the population-at-risk is those undergoing surgery and we adjusted for the proportion of those who were male, with White ethnicity, aged 65 years or older, living in the 20% of most deprived areas and with 2 or more Elixhauser comorbidities based on their admissions in the previous 24 months.

We performed a set of sensitivity analyses (Supplementary Table 2). Firstly, we ran placebo tests to check whether similar effects would be found in non-hub trusts as model-induced artifacts. Secondly, we controlled for the proportions of each HVLC specialty performed at each trust to reduce unobserved bias. Thirdly, we re-included the lockdown period in the analysis to check if the suspension of planned surgery at that time would skew effects. Finally, instead of examining hub activity using HVLC elective activity, we used an alternative data-driven approach to proxy hub activity (Supplementary Table 3).

Analyses were performed using the gsynth package (version 1.0.9, https://cran.r-project.org/web/packages/gsynth/index.html) in R (version 4.0.2).

### Reporting summary

Further information on research design is available in the Nature Portfolio Reporting Summary linked to this article.

## Supplementary information

Supplementary information

Reporting Summary

Transparent Peer Review file

## Source data

Source Data

## Data Availability

This work uses data from Hospital Episode Statistics (HES), which is provided by patients as part of their care and support. Individual patient-level data are supplied under a data sharing agreement with NHS England and cannot be made available by the study team due to ethical and legal constraints around sensitive patient data. HES data are available under restricted access for monitoring, evaluation, and research purposes (full list here: https://digital.nhs.uk/services/hospital-episode-statistics), and access can be obtained by applying through NHS England’s Data Access Request Service (https://digital.nhs.uk/services/data-access-request-service-dars). Source data for figures are provided with this paper. Source data are provided with this paper.

## Code availability

Code for our analysis is available on GitHub: Co, M., Marks, T., Tracey, F., Conti, S., & Clarke, G. M. *The impact of elective surgical hubs on elective surgery in acute hospital trusts in England*. IAU Elective Hubs evaluation v1.0.0. 10.5281/zenodo.15274589. 2025.
