# Supplementary material for: The impact of elective surgical hubs on elective surgery in acute hospital trusts in England
Source: Nat Commun. 2025 Jul 4;16:6192. doi: 10.1038/s41467-025-60936-6 (PMC12227523; doi:10.1038/s41467-025-60936-6)
Supplement: Supplementary file 1 — Supplementary information [file 41467_2025_60936_MOESM1_ESM.pdf]

The impact of elective surgical hubs on elective surgery in acute hospital trusts in England

Supplementary Material

Contents

Appendix 1 - Supplementary Tables 2
Supplementary Table 1. Procedures included for each High-Volume Low-Complexity specialty 2
Supplementary Table 2. Summary of robustness checks and sensitivity analyses 3
Supplementary Table 3. 50 most common procedures carried out at trusts with hubs during our study period 6
Supplementary Table 4. Estimated average effect of elective hubs on trust-wide elective activity by specialty between January 2019 and December 2022 7
Appendix 2 - Supplementary Figures 8
Supplementary Figure 1. Flowchart of inclusions and exclusions 9
Supplementary Figure 2. Opening of hubs across time 10
Supplementary Figure 3. Trends in (A) total elective and (B) HVLC elective day-case proportion between April 2019 and December 2022 in new-hub, established-hub and non-hub trusts in England .... **Error! Bookmark not defined.**
Supplementary Figure 4. Trends in (A) total elective surgery and (B) HVLC elective surgery inpatient length of hospital stay between April 2019 and December 2022 in new-hub, established-hub and non-hub trusts in England. 12
Supplementary Figure 5. Estimated effect of elective hubs on (A) total elective surgery and (B) HVLC elective surgery day-case proportion in new-hub trusts for 12 months pre- and post-opening for hubs opening from January 2019 and December 2022 in England. 13
Supplementary Figure 6. Estimated effect of elective hubs on (A) total elective surgery and (B) HVLC elective surgery inpatient length of stay in new-hub trusts for 12 months pre- and post-opening for hubs opening from January 2019 onwards in England. 14
Supplementary Figure 7. Estimated effect of elective hubs on (A) total elective surgery and (B) HVLC elective surgery day-case proportion in established-hub trusts between April 2018 and December 2022 in England 15
Supplementary Figure 8. Estimated effect of elective hubs on (A) total elective surgery and (B) HVLC elective surgery inpatient length of stay in established-hub trusts between April 2018 and December 2022 in England 16
Supplementary Figure 9. Forest plots of individual estimated effects at new-hub trusts... 17
Supplementary Figure 10. Forest plots of individual estimated effects at established-hub trusts 19
Appendix 3 – Supplementary Methods: The generalised synthetic control method (GSC) .. 21
Rationale for choosing the GSC method 21
Framework and GSC method 21
Diagnostics 22

References 24

Appendix 1 - Supplementary Tables

Supplementary Table 1. Procedures included for each High-Volume Low-Complexity specialty

Speciality	Procedures	Version	Link
Ear, nose and throat	Endo sinus surgery, Tonsillectomy, Myringoplasty, Septoplasty and Turbinate surgery, Septorhinoplasty	Version 5: 08/12/21	https://www.gettingitrightfirsttime.co.uk/wp-content/uploads/2021/12/ENT_2021-12-23_Coding_HVLC-pathway-coding-recipes.pdf
General surgery	Laparoscopic cholecystectomy,<br>Primary inguinal hernia repair,<br>Para-umbilical hernia	Version 5: 20/12/21	https://www.gettingitrightfirsttime.co.uk/wp-content/uploads/2021/12/General-surgery_2021-12-23_Coding_HVLC-pathway-coding-recipes.pdf
Gynaecology and maternity	Operative laparoscopy, Laparoscopic hysterectomy, Endometrial ablation, Hysteroscopy, Vaginal hysterectomy and/or vaginal wall repair	Version 3: 16/12/21	https://www.gettingitrightfirsttime.co.uk/wp-content/uploads/2021/12/Mat-Gynae_2021-12-23_Coding_HVLC-pathway-coding-recipes.pdf
Ophthalmology	Low complexity cataract surgery	Version 2: 03/12/21	https://www.gettingitrightfirsttime.co.uk/wp-content/uploads/2021/12/Ophthalmology_2021-12-23_Coding_HVLC-Pathway-Coding-recipes.pdf
Orthopaedics	Anterior cruciate ligament reconstruction, Total hip replacement, Total knee replacement, Uni knee replacement, Bunions, Therapeutic shoulder arthroscopy	Version 2: 24/09/2021	https://www.gettingitrightfirsttime.co.uk/wp-content/uploads/2021/12/Orthopaedic-Elective_2021-12-23_Coding_HVLC-pathway-coding-recipes.pdf
Spinal	Lumbar Decompression / Discectomy, Cervical spine decompression / fusion, Lumbar medial branch block/facet joint injections, Lumbar nerve root block / therapeutic epidural injection, One or two level posterior fusion surgery (PLF, TLIF, PLIF)	Version 6: 20/12/2021	https://www.gettingitrightfirsttime.co.uk/wp-content/uploads/2021/12/Spinal-Surgery_2021-12-23_Coding_HVLC-pathway-coding-recipes.pdf
Urology	Bladder outflow obstruction, Bladder tumour resection (TURBT), Cystoscopy plus, Ureteroscopy and stent management, Minor peno-scrotal surgery	Version 2: 03/12/2021	https://www.gettingitrightfirsttime.co.uk/wp-content/uploads/2021/12/Urology_2021-23-12_Coding_HVLC-Pathway-Coding-recipes.pdf

Spinal surgeries were excluded from these because we did not have information on which trusts offered spinal surgery procedures

Supplementary Table 2. Summary of robustness checks and sensitivity analyses

Sensitivity analysis	Rationale	Method	Effect estimate(95% CI)	Result	Impact on main analysis
**Placebo test**	Test plausibility of core assumptions of the generalised synthetic control method. Method should not find significant estimates of effect in non-hub trusts.	We estimated the effect of elective hubs in placebo groups without an elective hub. We created 5 placebo groups comprising mutually exclusive sets of 11 non-hub trusts each. For each placebo group we repeated the same analysis for established-hub trusts for outcomes total and HVLC elective activity.	Established-hub trusts:<br>Placebo Group 1<br>Total activity: 0.059 (-0.416 to 0.534)<br>HVLC activity: -0.053(-0.161 to 0.055)<br>Placebo Group 1<br>Total activity: 0.060 (-0.342 to 0.463)<br>HVLC activity: -0.019 (-0.117 to 0.079)<br>Placebo Group 1<br>Total activity: -0.039 (-0.582 to 0.504)<br>HVLC activity: -0.026 (-0.146 to 0.094)<br>Placebo Group 1<br>Total activity: -0.042 (-0.458 to 0.374)<br>HVLC activity: -0.009 (-0.079 to 0.098)<br>Placebo Group 1<br>Total activity: -0.083 (-0.551 to 0.386)<br>HVLC activity: -0.106 (-0.004 to 0.208)	None of the models found significant effects in the placebo groups.	Results from the main analysis are unlikely to be due to method assumptions.
**Controlling for specialties**	Assess whether differences in the mix of specialties offered by each trust or changes to these specialty mixes over time affects the results	We calculated the proportion of HVLC activity comprised by each specialty (e.g. {number of orthopaedic procedures} / {number of HVLC procedures}) for each month. We estimated effects for new- and established-hub trusts for total activity these proportions as covariates in the model.	New-hub trusts:<br>Total activity: 0.233 (-0.028 to 0.493)<br><br>Established-hub trusts:<br>Total activity: 0.035 (-0.295 to 0.365)	Effect sizes were consistent with the main analysis of new-hub trusts. However, for established-hub trusts, the higher rate of total elective activity was no longer significant when controlling for specialty mix compared with the main analysis.<br><br>No specialty covariate coefficient was estimated to be significant in these models.	Results remain largely consistent when controlling for changes in the specialty mix over time. Additional covariates did not significantly add predictive power to the main analysis models, supporting the decision to drop them.

Sensitivity analysis	Rationale	Method	Effect estimate(95% CI)	Result	Impact on main analysis
**Including COVID-19 lockdown period**	To assess the impact of removing the COVID-19 period where elective activity was restricted by government mandate.	We estimated effects for new- and established- hub trusts for total and HVLC elective activity without excluding the period from April 2020 to March 2021.	New-hub trusts:<br>Total activity: 0.140 (-0.093 to 0.372)<br>HVLC activity: 0.137 (0.063 to 0.211)<br><br>Established-hub trusts:<br>Total activity: 0.374 (0.043 to 0.705)<br>HVLC activity: 0.101(0.016 to 0.186)	Results for established-hub trusts were consistent with the main analysis results.<br><br>For both new and established-hub trusts, the estimated number of latent factors indicated poorer model fit compared with the main analysis (5 vs 4 latent factors respectively).	Elective activity rates were no longer significant in new-hub trusts, but model fit was also worse: the lockdown introduced volatility that was difficult to model.
		We estimated effects for new-hub trusts only for total and HVLC elective activity extending the lockdown excluded period by one month either side so that the exclusion period was March 2020 to April 2021	New-hub trusts:<br>Total activity: 0.143 (-0.099 to 0.386)<br>HVLC activity: 0.136 (0.061 to 0.211)<br><br>Established-hub trusts:<br>Total activity: 0.438 (0.043 to 0.834)<br>HVLC activity: 0.107 (0.011 to 0.203)	Results were consistent with the main analysis results.	The exclusion of March 2020 and April 2021 did not affect the impact assessment on total activity in new-hub trusts, suggesting that any anticipatory or tapering effect on total activity of the COVID-19 lockdown did not significantly affect counterfactual and effect estimates.

Sensitivity analysis	Rationale	Method	Effect estimate(95% CI)	Result	Impact on main analysis
**Alternative proxy for hub activity**	In the main analysis we use HVLC activity as a proxy for activity most likely to be taking place in the hub. As an alternative, we also look at the most common procedures being performed across a trust. This was to investigate whether an alternative definition to proxy hub activity significantly changed results.	We identified the 50 most common procedures being performed across all trusts (see Supplementary Table 2.1). We then used these procedures to define a subset of elective activity which we refer to here as high-volume. We estimated effects for new- and established-hub trusts for this subset	Established-hub trusts:<br>High-volume activity: 0.267 (0.098 to 0.437)<br><br>New-hub trusts:<br>High-volume: 0.145 (0.003 to 0.288)	Effects estimates were higher and with wider confidence intervals for established-hub trusts using high-volume activity compared with the main analysis using HVLC activity.<br><br>Effect estimates were slightly lowe and with wider confidence intervals for new-hub trusts using high-volume activity compared with the main analysis using HVLC activity.	Estimates were similar when looking at high-volume activity compared to HVLC activity, supporting findings that activity rates for high-volume procedures (using either definition) increased in both new- and established-hub trust.

Supplementary Table 3. 50 most common procedures carried out at trusts with hubs during our study period

OPCS-4 code	Procedure
X292	Continuous intravenous infusion of therapeutic substance NEC
C751	Insertion of prosthetic replacement for lens NEC
G451	Fibreoptic endoscopic examination of upper gastrointestinal tract and biopsy of lesion of upper gastrointestinal tract
W903	Injection of therapeutic substance into joint
W401	Primary total prosthetic replacement of knee joint using cement
H229	Unspecified diagnostic endoscopic examination of colon
H221	Diagnostic fibreoptic endoscopic examination of colon and biopsy of lesion of colon
M459	Unspecified diagnostic endoscopic examination of bladder
X281	Intermittent intravenous infusion of therapeutic substance
H201	Fibreoptic endoscopic snare resection of lesion of colon
X723	Delivery of simple parenteral chemotherapy for neoplasm at first attendance
G459	Unspecified diagnostic fibreoptic endoscopic examination of upper gastrointestinal tract
X721	Delivery of complex chemotherapy for neoplasm including prolonged infusional treatment at first attendance
C794	Injection into vitreous body NEC
A735	Injection of therapeutic substance around peripheral nerve
S069	Unspecified other excision of lesion of skin
X384	Subcutaneous chemotherapy
H259	Unspecified diagnostic endoscopic examination of lower bowel using fibreoptic sigmoidoscope
X362	Venesection
A651	Carpal tunnel release
M702	Perineal needle biopsy of prostate
U051	Computed tomography of head
U212	Computed tomography NEC
X724	Delivery of subsequent element of cycle of chemotherapy for neoplasm
W381	Primary total prosthetic replacement of hip joint not using cement
S065	Excision of lesion of skin of head or neck NEC
X722	Delivery of complex parenteral chemotherapy for neoplasm at first attendance
W941	Primary hybrid prosthetic replacement of hip joint using cemented femoral component
T202	Primary repair of inguinal hernia using insert of prosthetic material
J183	Total cholecystectomy NEC
A577	Injection of therapeutic substance around spinal nerve root
X332	Intravenous blood transfusion of packed cells
V544	Injection around spinal facet of spine
W283	Removal of internal fixation from bone NEC
W822	Endoscopic resection of semilunar cartilage NEC
H251	Diagnostic endoscopic examination of lower bowel and biopsy of lesion of lower bowel using fibreoptic sigmoidoscope
M473	Removal of urethral catheter from bladder
X731	Delivery of exclusively oral chemotherapy for neoplasm
X369	Unspecified blood withdrawal
B282	Partial excision of breast NEC
H207	Fibreoptic endoscopic mucosal resection of lesion of colon
Q181	Diagnostic endoscopic examination of uterus and biopsy of lesion of uterus
W371	Primary total prosthetic replacement of hip joint using cement
Q171	Endoscopic resection of lesion of uterus
M479	Unspecified urethral catheterisation of bladder
W742	Reconstruction of intra-articular ligament NEC
R249	All normal delivery
U201	Transthoracic echocardiography
M494	Introduction of therapeutic substance into bladder
X729	Unspecified delivery of chemotherapy for neoplasm

Supplementary Table 4. Estimated average effect of elective hubs on trust-wide elective activity by specialty between January 2019 and December 2022

HVLC specialty	Percentage of HVLC activity across all trusts	Coefficient	95% CI	P-value
**New-hub trusts**				
Elective activity rate (per 1,000 trust catchment population per month)				
Ear, Nose and Throat	6%	0.007	-0.003 to 0.017	0.168
Gynaecology	10%	0.007	-0.007 to 0.020	0.327
General surgery	13%	0.023	0.007 to 0.039	0.005*
Orthopaedic	16%	0.009	-0.040 to 0.059	0.713
Urology	17%	-0.002	-0.025 to 0.021	0.863
Ophthalmology	30%	0.081	-0.001 to 0.163	0.054
Spinal	8%	-	-	-
**Established-hub trusts**				
Elective activity rate (per 1,000 trust catchment population per month)				
Ear, Nose and Throat	4%	0.011	-0.002 to 0.024	0.099
Gynaecology	8%	0.001	-0.015 to 0.017	0.933
General surgery	11%	0.016	-0.001 to 0.033	0.073
Orthopaedic	20%	0.059	-0.023 to 0.140	0.159
Urology	13%	-0.001	-0.034 to 0.032	0.938
Ophthalmology	32%	0.021	-0.058 to 0.100	0.605
Spinal	12%	-	-	-

For new-hub trusts, coefficients are the estimated average effect of opening a hub during the first 12 months of opening between January 2019 and December 2022 excluding the UK government COVID-19 lockdown period between April 2020 and March 2021. For established-hub trusts, coefficients are the estimated effect of an established hub on elective activity after the end of the UK government COVID-19 lockdown in April 2021 until December 2022. Estimates are from the generalised synthetic control model. 95% confidence intervals and p-values are derived from non-parametric bootstrap pairwise difference two-sided T tests, unadjusted for multiple comparisons. Effect estimates are statistically significant at * $p < 0.05$.

Appendix 2 - Supplementary Figures

Supplementary Figure 1. Flowchart of inclusions and exclusions

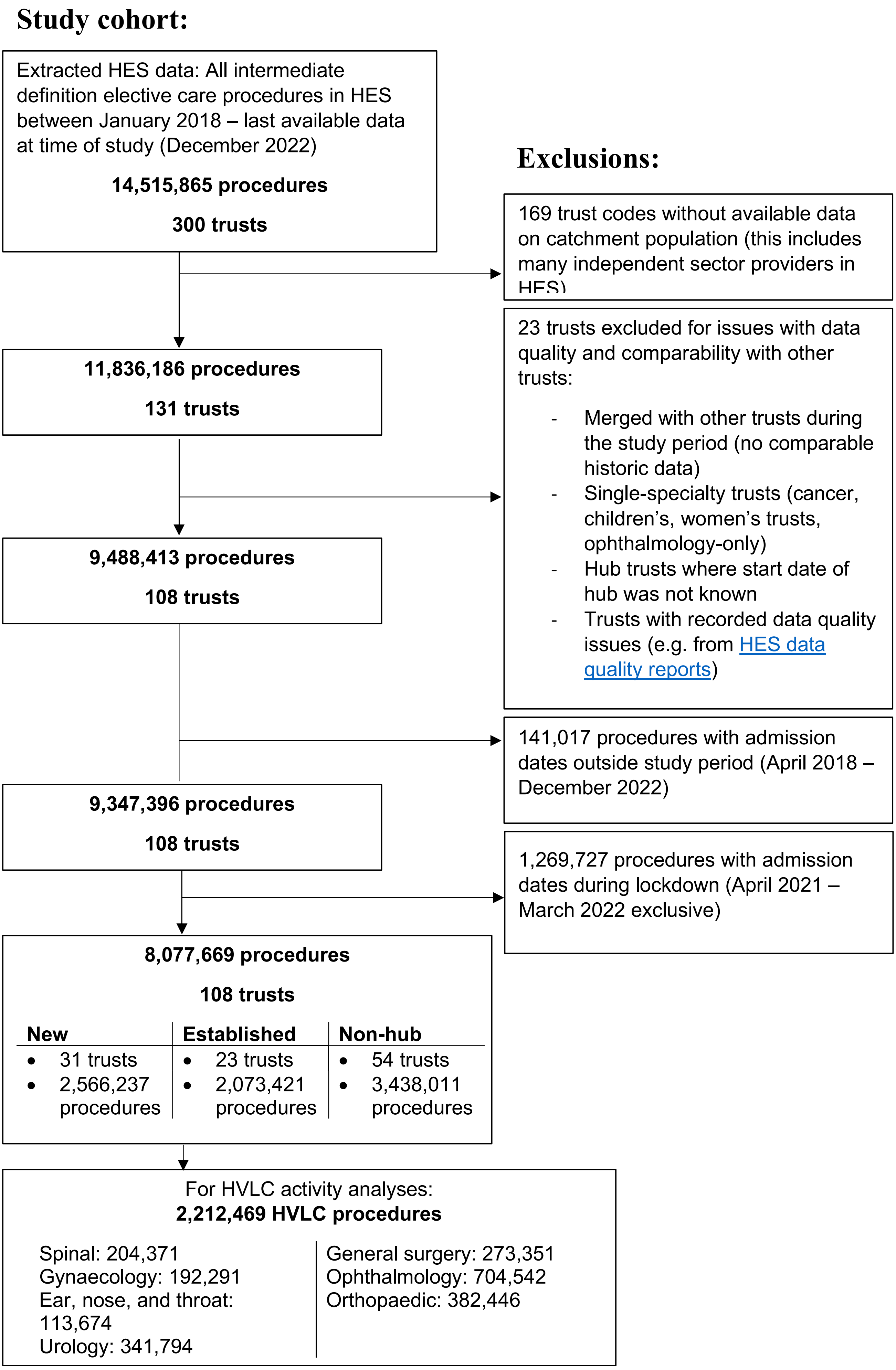

Supplementary Figure 2. Opening of hubs across time

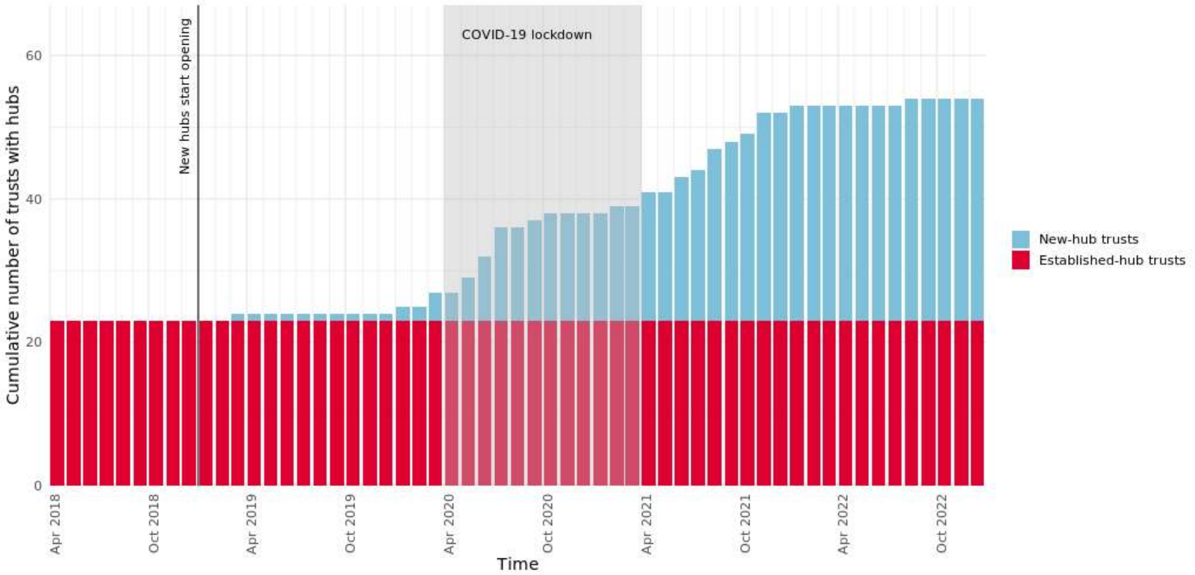

Plot shows monthly cumulative total number of trusts in our study with at least one hub. Hubs opening before January 2019 are defined as established hubs. Hubs opening from January 2019 onwards are defined as new hubs. By January 2019 there were 23 established-hub trusts. By March 2020 (prior to the lockdown period, shaded in grey), there were 27 hub trusts (23 established-hub and 4 new-hub trusts). By the end of the lockdown period in April 2021, there were 41 hub trusts (23 established-hub and 18 new-hub trusts). At the end of our study period (October 2022) there were 54 hub trusts.

Supplementary Figure 3. Trends in (A) total elective and (B) HVLC elective day-case proportion between April 2019 and December 2022 in 31 new-hub, 23 established-hub and 54 non-hub trusts in England

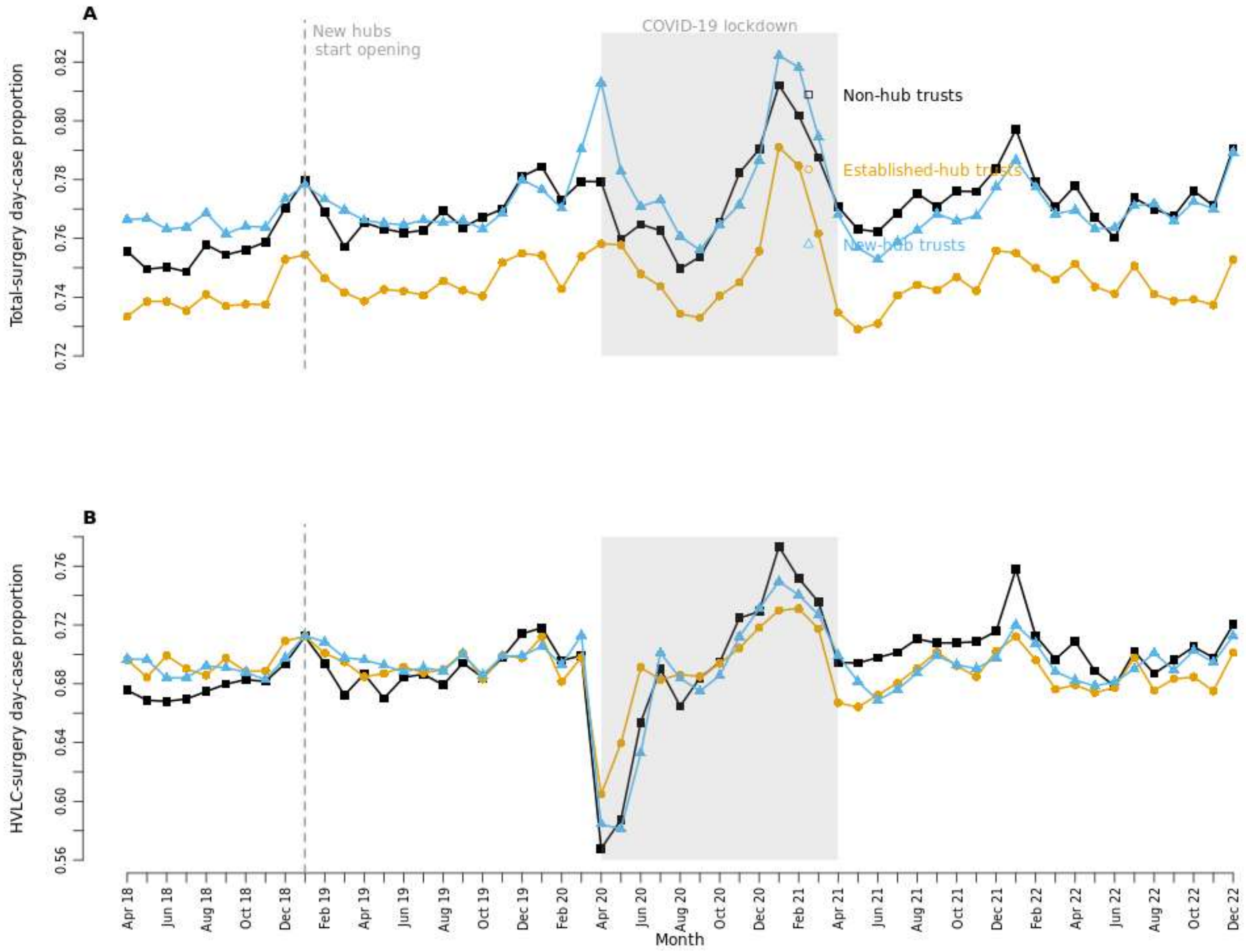

Grey shaded area indicates timing of the UK government COVID-19 lockdown period between April 2020 and March 2021. Dashed vertical lines indicate the timepoint for the opening of new hub trusts.

Supplementary Figure 4. Trends in (A) total elective surgery and (B) HVLC elective surgery inpatient length of hospital stay between April 2019 and December 2022 in 31 new-hub, 23 established-hub and 54 non-hub trusts in England.

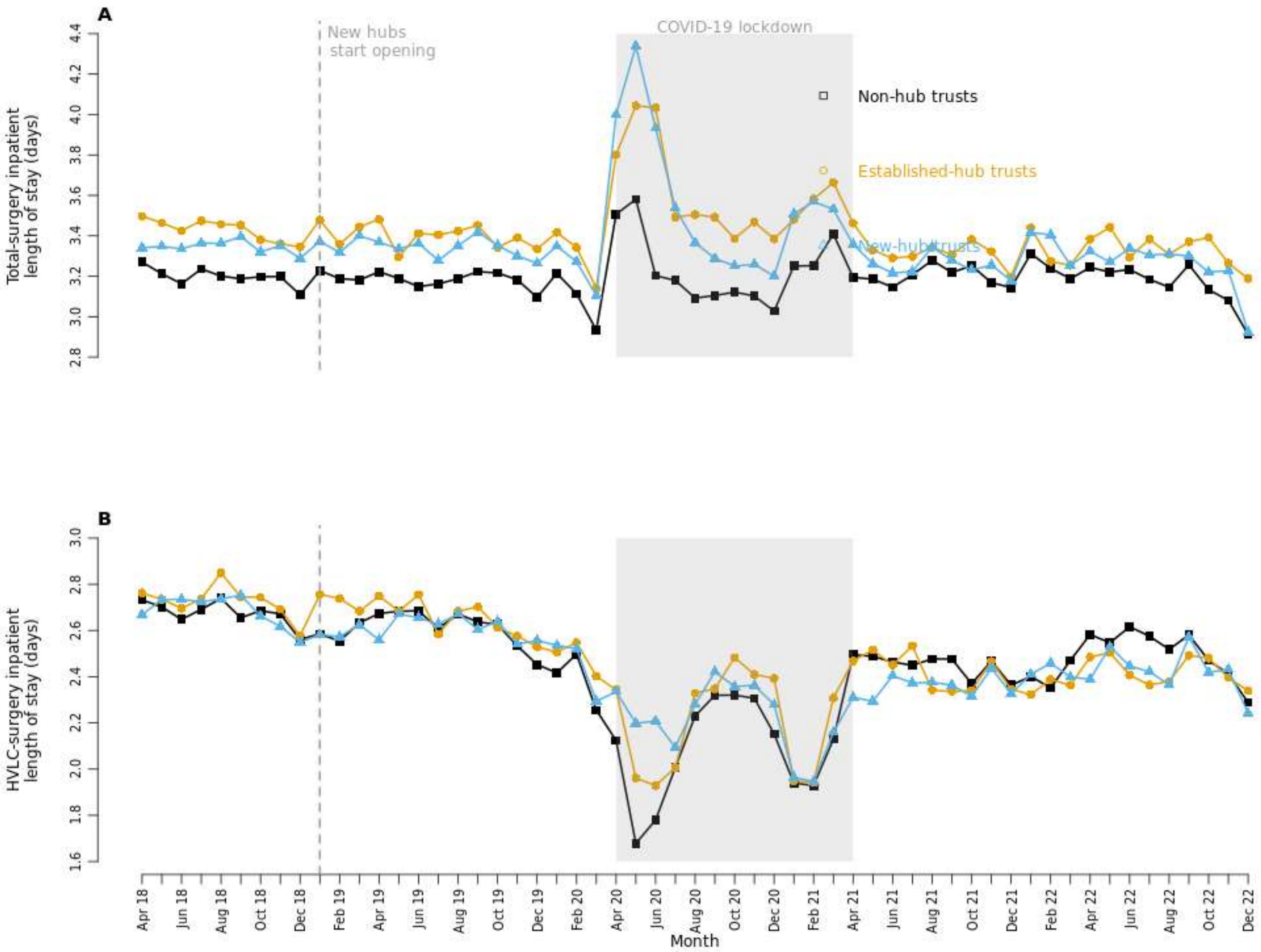

Grey shaded area indicates timing of the UK government COVID-19 lockdown period between April 2020 and March 2021. Dashed vertical lines indicate the timepoint for the opening of new hub trusts.

Supplementary Figure 5. Estimated effect of elective hubs on (A) total elective surgery and (B) HVLC elective surgery day-case proportion in 31 new-hub trusts for 12 months pre- and post-opening for hubs opening from January 2019 and December 2022 in England. Results exclude the UK government COVID-19 lockdown period between April 2020 and March 2021. Estimates are derived from the generalised synthetic control model. Dashed vertical lines indicate the timepoint for the opening of new hub trusts. Shaded areas show non-parametric bootstrap 95% confidence intervals around effect estimates.

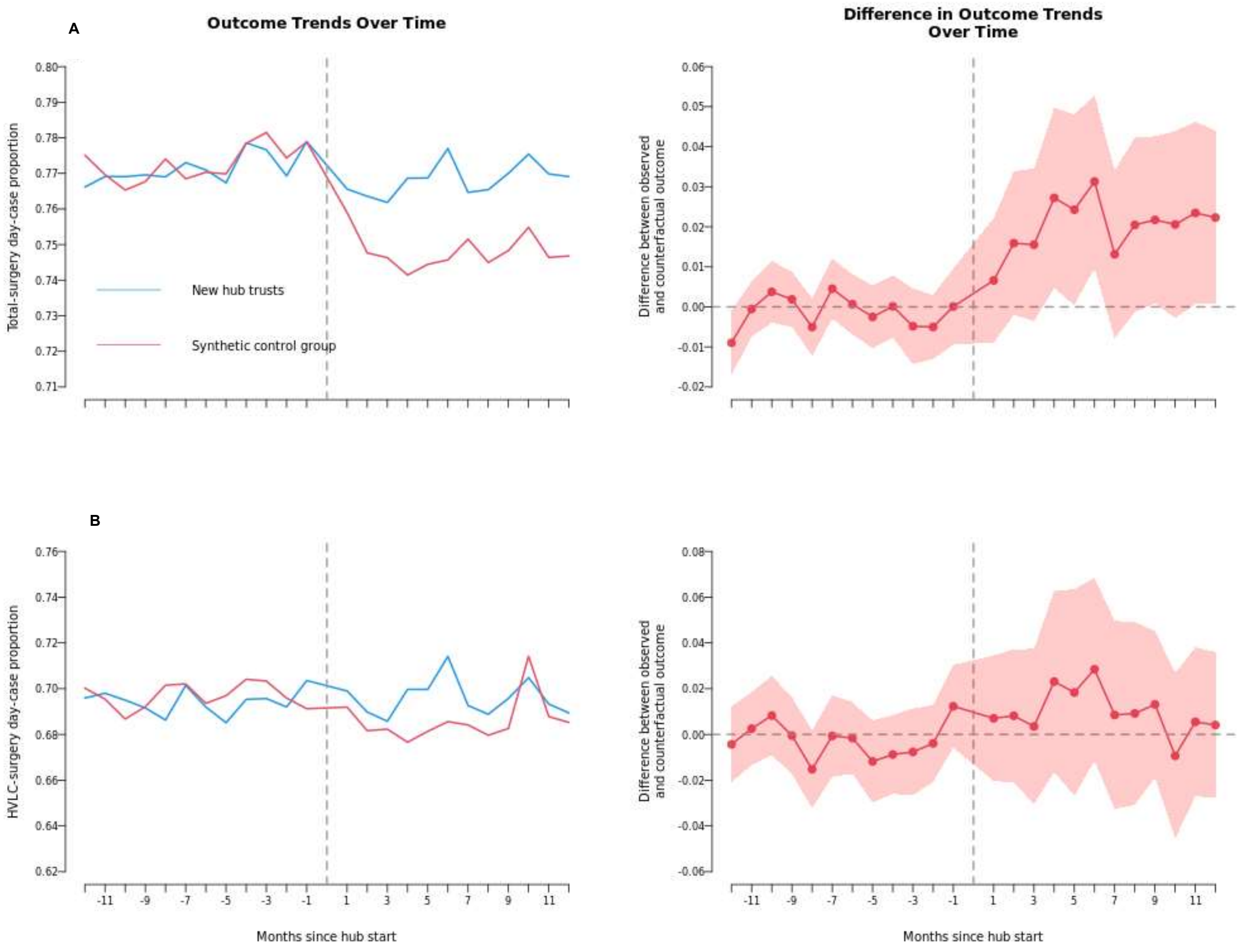

Supplementary Figure 6. Estimated effect of elective hubs on (A) total elective surgery and (B) HVLC elective surgery inpatient length of stay in 31 new-hub trusts for 12 months pre- and post-opening for hubs opening from January 2019 onwards in England. Results exclude the UK government COVID-19 lockdown period between April 2020 and March 2021. Estimates are derived from the generalised synthetic control model. Dashed vertical lines indicate the timepoint for the opening of new hub trusts. Shaded areas show non-parametric bootstrap 95% confidence intervals around effect estimates.

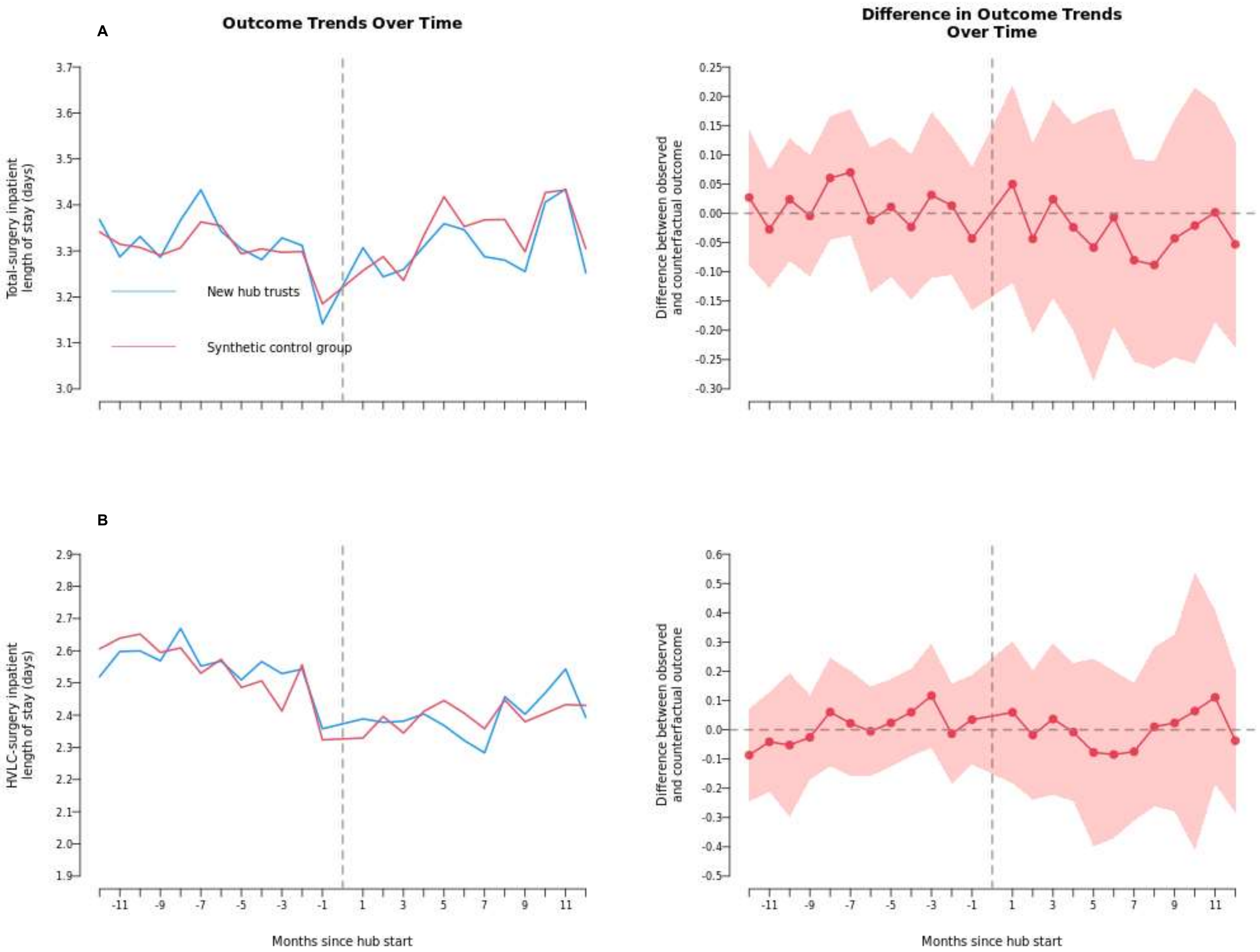

Supplementary Figure 7. Estimated effect of elective hubs on (A) total elective surgery and (B) HVLC elective surgery day-case proportion in 23 established-hub trusts between April 2018 and December 2022 in England. Results exclude the UK government COVID-19 lockdown period between April 2020 and March 2021. Estimates are derived from the generalised synthetic control model. Dashed vertical lines indicate the timepoint for the start of the analysis. Shaded areas show non-parametric bootstrap 95% confidence intervals around effect estimates.

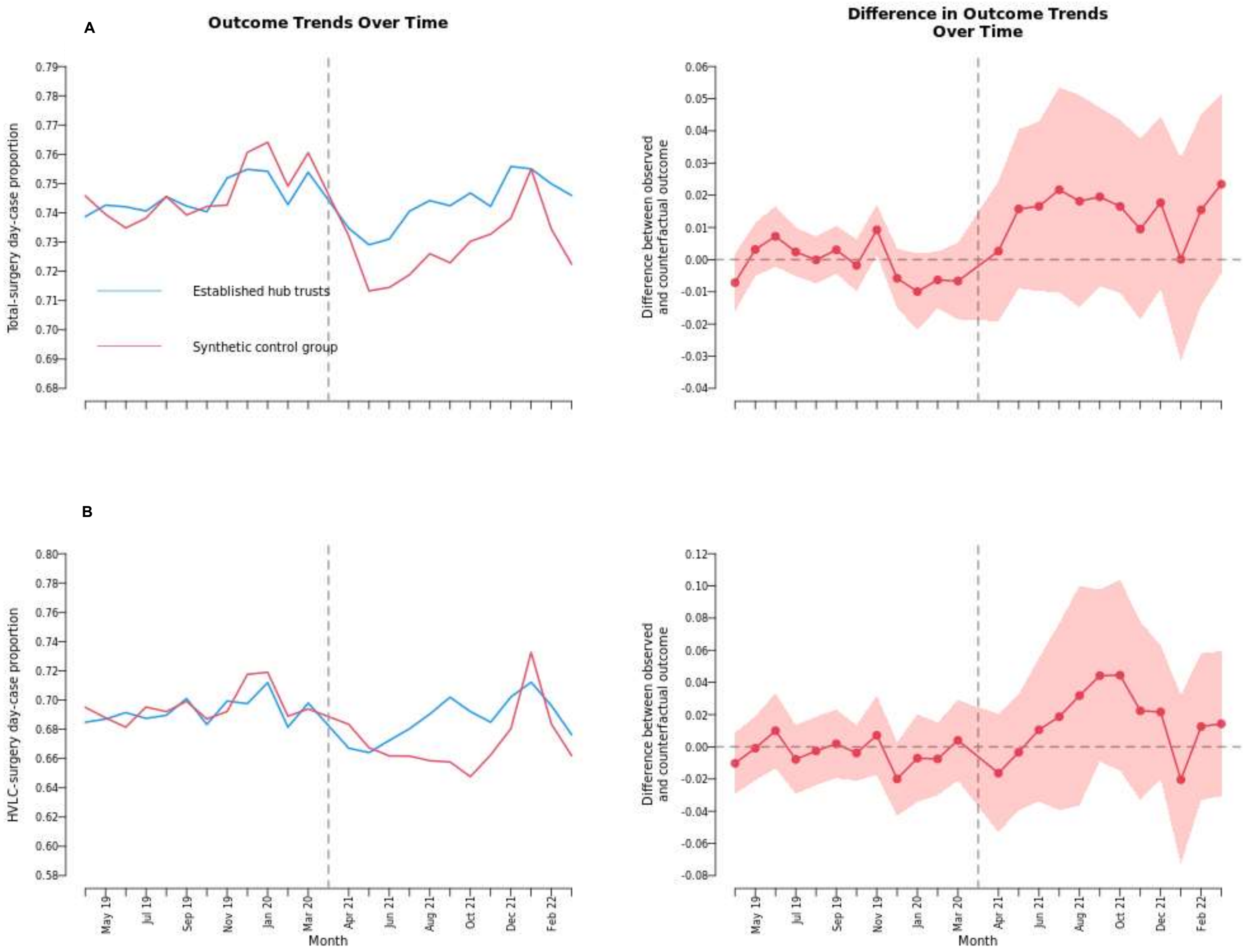

Supplementary Figure 8. Estimated effect of elective hubs on (A) total elective surgery and (B) HVLC elective surgery inpatient length of stay in 23 established-hub trusts between April 2018 and December 2022 in England. Results exclude the UK government COVID-19 lockdown period between April 2020 and March 2021. Estimates are derived from the generalised synthetic control model. Dashed vertical lines indicate the timepoint for the start of the analysis. Shaded areas show non-parametric bootstrap 95% confidence intervals around effect estimates.

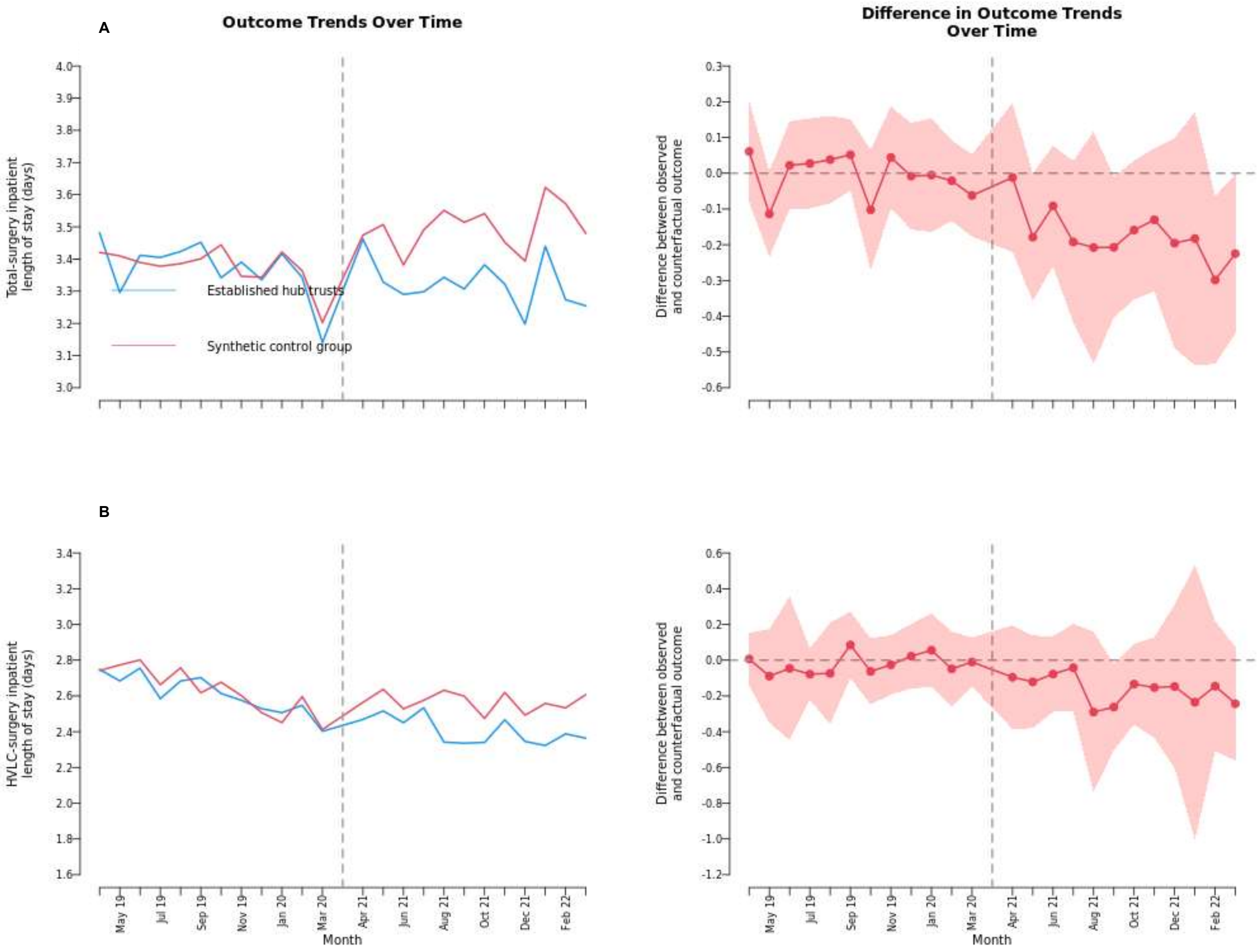

Supplementary Figure 9. Forest plots of individual estimated effects at new-hub trusts. Individual trust estimated effects of a new elective hub (opening from January 2019 onwards) on A. total and B. HVLC elective surgery rate per 1,000 trust catchment population in the first 12 months of opening. Analyses exclude the COVID-19 lockdown period between April 2020 and March 2021. Results were obtained using the generalised synthetic control model. Trust names have been pseudonymised with a number between 1-31. Estimated effect sizes and 95% confidence intervals are shown. Confidence intervals are derived using parametric bootstrap procedures. The overall model estimate is indicated at the bottom of the plot by an open diamond and as a dotted vertical line for comparison across trusts. The solid vertical line indicates no effect. Statistically significant individual estimates should be interpreted with caution as the large number of estimates increases the risk of observing a significant result by chance.

A. Total elective surgery rate

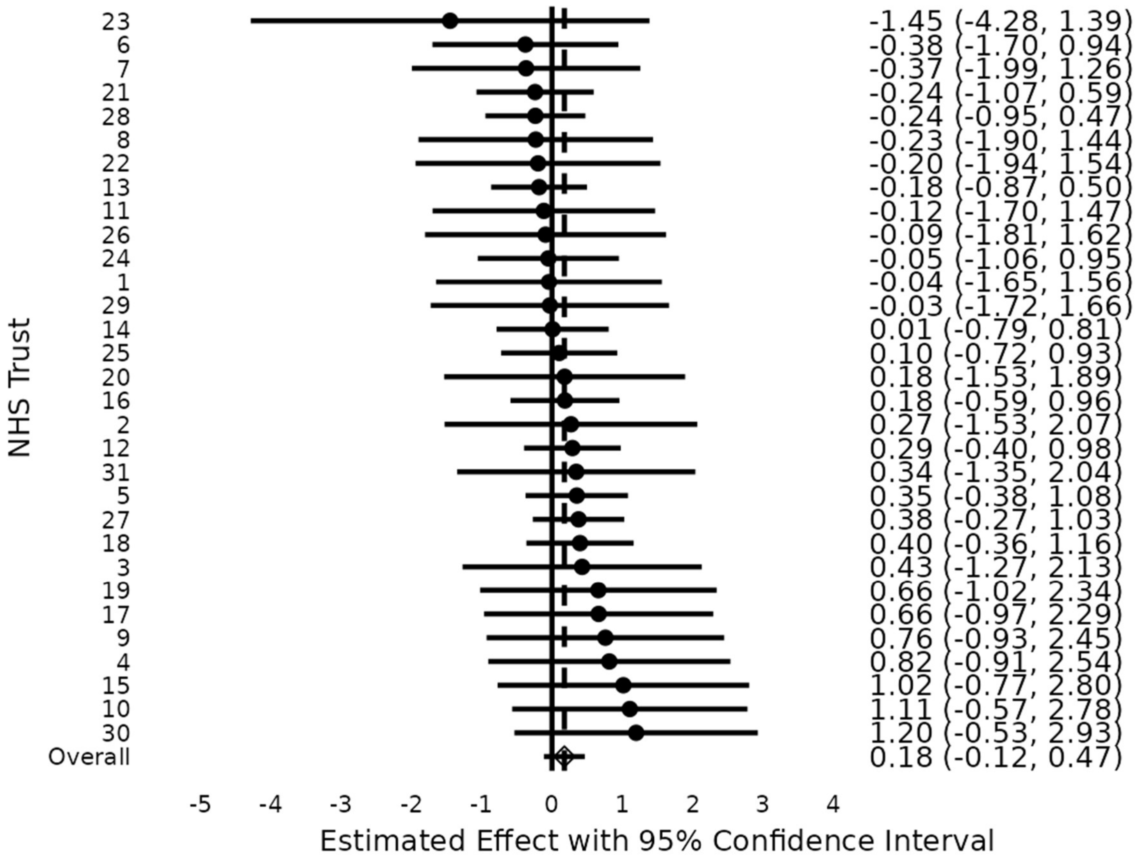

B. HVLC elective surgery rate

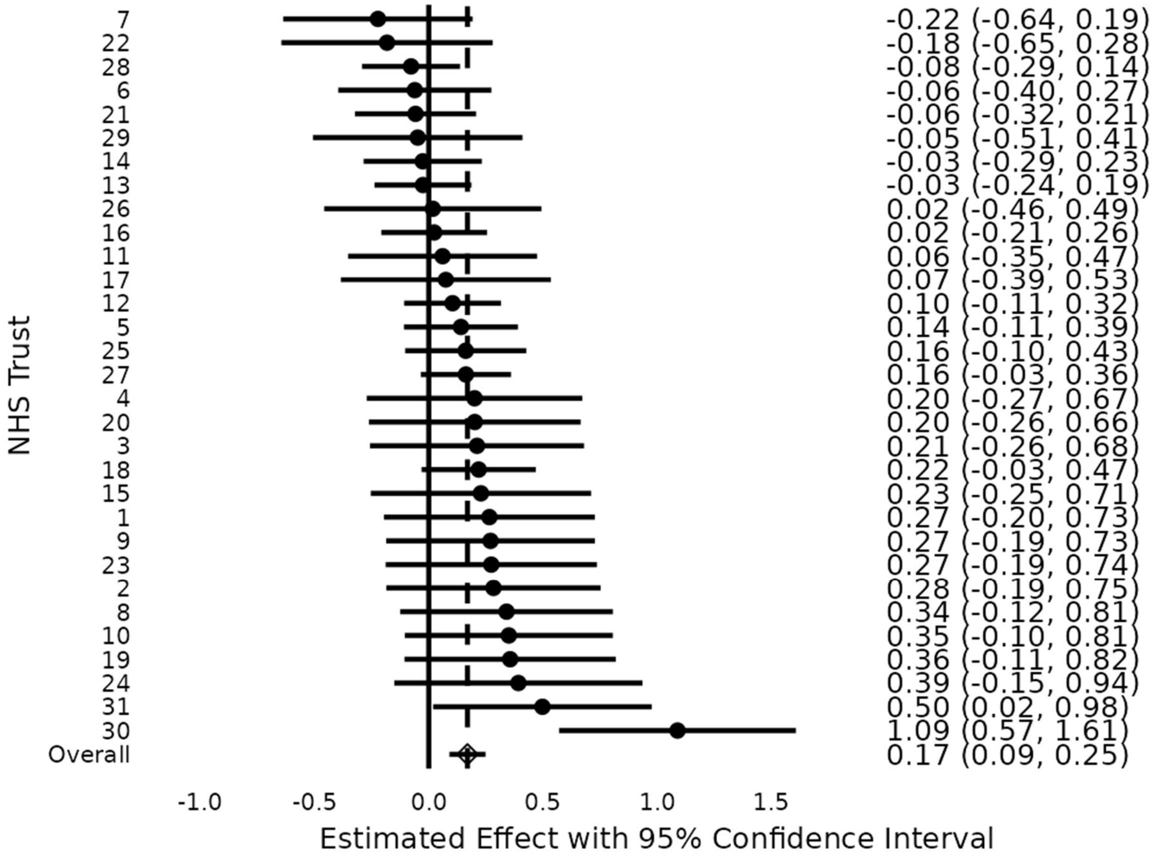

Supplementary Figure 10. Forest plots of individual estimated effects at established-hub trusts. Individual estimated effects of a having an established hub (opening before January 2019) on A. total and B. HVLC elective surgery rate per 1,000 trust catchment population from April 2021 to December 2022. Results were obtained using the generalised synthetic control model. Trust names have been pseudonymised with a number between 1-23. Estimated effect sizes and 95% confidence intervals are shown. Confidence intervals are derived using parametric bootstrap procedures. The overall model estimate is indicated at the bottom of the plot by an open diamond and as a dotted vertical line for comparison across trusts. The solid vertical line indicates no effect. Statistically significant individual estimates should be interpreted with caution as the large number of estimates increases the risk of observing a significant result by chance.

A. Total elective surgery rate

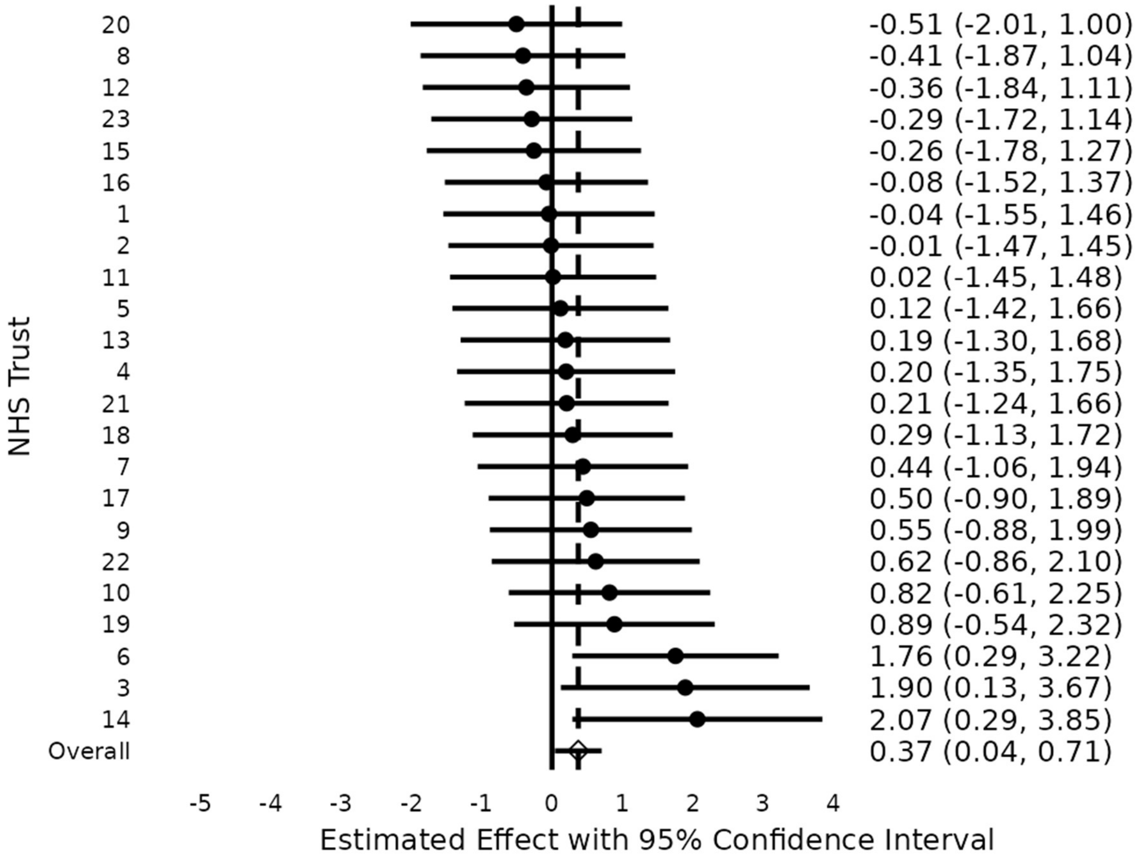

B. HVLC elective surgery rate

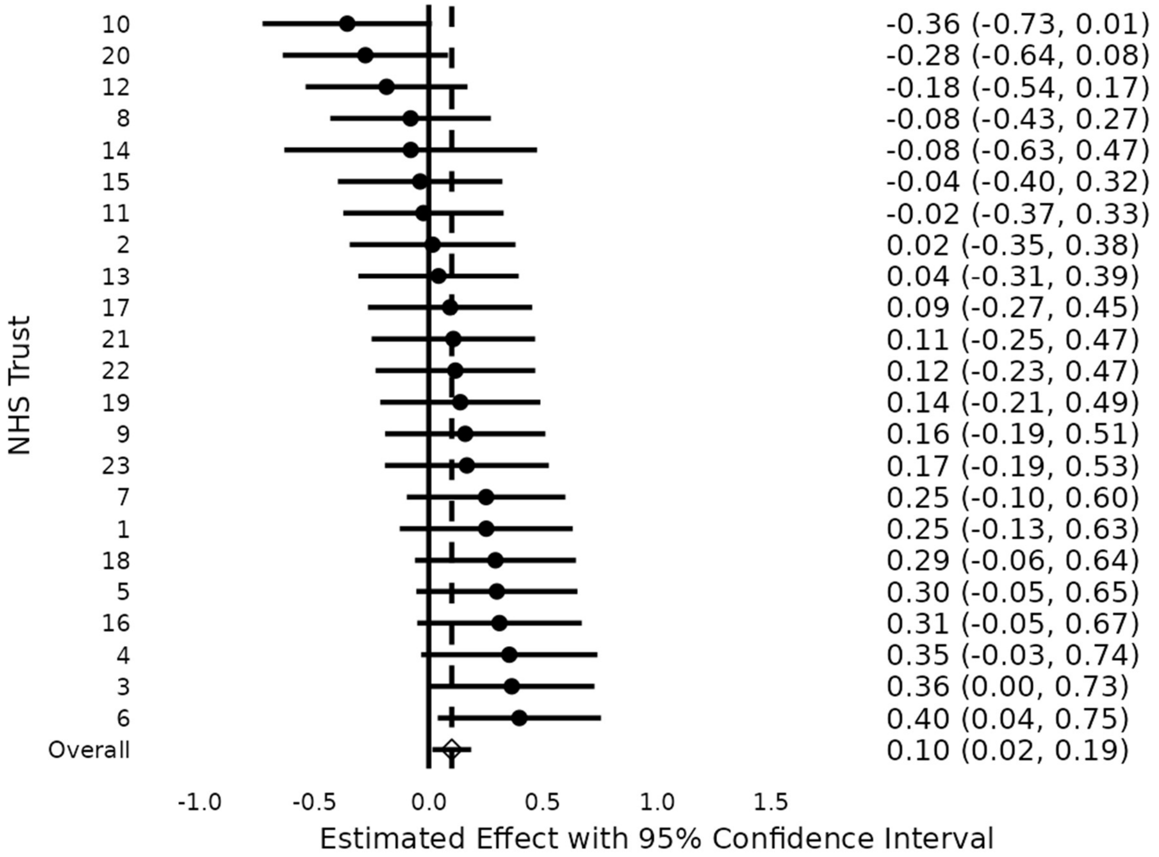

Appendix 3 – Supplementary Methods: The generalised synthetic control method

Rationale for choosing the method

The generalised synthetic control method (GSC) approach[1] is suitable for assessing the impact of a health care intervention (in this case having a hub) where: (i) the data are observational, so that allocation to treatment (trust with a hub) or control (trust without a hub) is not random but influenced by other factors; (ii) the treatment is binary (trusts either have hubs or they do not); (iii) there are multiple treated units and varying treatment periods (trusts opened hubs at different times); and, (iv) outcome measurements are available at multiple time points before and after the intervention is introduced, thus giving rise to panel data[2,3].

A main advantage of the GSC approach is that it does not rely on the restrictive 'parallel trends assumption' required by the widely used difference-in-difference approach, which is implausible in many health policy contexts. Instead, the GSC method assumes 'strict exogeneity, or more simply that model residuals are independent of treatment assignment, observed covariates, latent factors (unobserved confounders) and their loadings. This is a key advantage of the GSC method accounting for unique aspects of typical health care data, namely the likely presence of unmeasured confounders and effects that vary across time and units. To meet this assumption, GSC incorporates unit-specific intercepts and time-varying coefficients to account for unobserved factors that may change over time and differ across units. Furthermore, GSC leverages information from treated units in the pre-intervention period to efficiently construct counterfactual outcomes.

Framework and GSC method

We assume there are *T* time periods (T_o before the intervention), and *I* units (the first I_o are untreated). Let Y_{it} represent one of *M* observed outcomes for unit *i* at time *t* and D_{it} a treatment indicator (D_{it} =1 if unit *i* is treated at time *t* and 0 otherwise). Let $Y_{it}(0)$ and $Y_{it}(1)$ be the potential outcomes in unit *i* at time *t* when $D_{it} = 0$ and $D_{it} = 1$ respectively. The observed outcome can be written as

$$Y_{it} = (1 - D_{it})Y_{it}(0) + D_{it}Y_{it}(1)$$

The generalised synthetic control (GSC) method assumes that Y_{it} can be represented by an interactive fixed effect regression model with the following linear factor structure:

$$Y_{it} = \delta_{it}D_{it} + \boldsymbol{x}'_{it}\boldsymbol{\beta} + \boldsymbol{\lambda}'_i\boldsymbol{f}_t + \varepsilon_{it} \ ,$$

where δ_{it} denotes the unit- and time-specific treatment effect; $\boldsymbol{x}_{it}$ a vector of k observed covariates with corresponding regression coefficients k-vector $\boldsymbol{\beta}$; $\boldsymbol{f}_t$ a vector of r unit-invariant unobserved (latent) factors with corresponding unknown time-invariant loadings $\boldsymbol{\lambda}_i$; and $\varepsilon_{it} \sim \mathcal{N}(0, \sigma^2)$ is a Normally distributed residual term with zero mean and constant variance σ^2.

The average treatment effect on the treated (ATT) over post-intervention time $t > T_0$ is defined as

$$ATT_{t,t>T_o} \coloneqq \hat{\delta}_t = \bar{Y}_t(1) - \bar{Y}_t(0)$$

where $\bar{Y}_t(1)$ and $\bar{Y}_t(0)$ are the average potential outcomes for the treated units in the presence and absence of treatment, respectively.

GSC model estimation is carried out in a three-step process:

1. In the first step, an interactive fixed effects model is fitted to observations from control units to obtain parameter estimates $\hat{\boldsymbol{\beta}}$, latent factors, $\hat{\boldsymbol{f}}_t$, and latent factor loadings $\hat{\boldsymbol{\lambda}}_i$ for the control units only.
2. In the second step, the remaining latent factor loadings for treated units are estimated by minimizing the mean squared error between the observed treated units' outcomes and those predicted by the interactive fixed effects model in the pre-intervention period.
3. The counterfactual for the treated unit $\hat{Y}_{it}(0)$ is then constructed based on $\hat{\boldsymbol{\beta}}$, $\hat{\boldsymbol{f}}_t$, and $\hat{\boldsymbol{\lambda}}_i$:

   $$\hat{Y}_{it}(0) = \boldsymbol{x}'_{it}\hat{\boldsymbol{\beta}} + \hat{\boldsymbol{\lambda}}'_i\hat{\boldsymbol{f}}_t \quad i > I_o, t > T_o$$

   and hence an estimate of the ATT for $t > T_o$ is:

   $$\hat{\delta}_t = \frac{1}{I - I_o}\sum_{i=I_0+1}^{I}\left(Y_{it}(1) - \hat{Y}_{it}(0)\right)$$

To ensure consistent identification of $\hat{\delta}_t$, the GSC approach assumes the regression errors ε_{it} are independent of treatment assignment, all observed covariates, latent factors and loadings: that is $\varepsilon_{it} \perp D_{js}, \boldsymbol{x}_{js}, \boldsymbol{\lambda}_j, \boldsymbol{f}_s \;\forall\, \mathrm{i,j,t,s}$. In addition, error terms are assumed to be at best weakly correlated over time for the same unit and uncorrelated at any time for different units. Bootstrap procedures are used to obtain uncertainty estimates of the GSC estimator. For more details on the approach and assumptions please refer to Xu[1].

For our application the treated units comprise either all established-hub trusts or all new-hub trusts. The control units comprise all non-hub trusts. We fitted a separate GSC model for all established-hub trusts simultaneously and for all new-hub trusts simultaneously for each outcome in turn. Analyses were performed using the 'gsynth' package (version 1.0.9, https://cran.r-project.org/web/packages/gsynth/index.html) in R (version 4.0.2). To obtain more interpretable impact estimates specifically from the analysis of new-hub trusts, we developed bespoke code to post-process output produced by the 'gsynth' package[4]. This software is freely available and documented at the team's institutional GitHub repository (URL: https://github.com/HFAnalyticsLab/elective-hubs/tree/main). Our code derives impact estimates as unweighted averages of trust- and month-specific effect estimates after these are temporally realigned relative to months lapsed since the hub opening, as opposed to calendar month.

Diagnostics

In order to gauge robustness of effect estimates, we examined the latent factors and loadings estimated by the GSC model. Models estimating more than 3 latent factors, or where the distribution of the treated unit factor loadings did not fall within the 10th and 90th percentile of the control unit factor loadings, were rejected due to evidence of extrapolation[3].

In order to assess good pre-intervention fit, we rejected models with a statistically significant difference between estimated and observed outcomes during the pre-intervention period.

References

1 Xu Y. Generalized Synthetic Control Method: Causal Inference with Interactive Fixed Effects Models. *Political Analysis* 2017; **25**. DOI:10.1017/pan.2016.2.

2 O'Neill S, Kreif N, Sutton M, Grieve R. A comparison of methods for health policy evaluation with controlled pre-post designs. *Health Serv Res* 2020. DOI:10.1111/1475-6773.13274.

3 Samartsidis P, Seaman SR, Presanis AM, Hickman M, De Angelis D. Assessing the causal effect of binary interventions from observational panel data with few treated units. *Statistical Science* 2019. DOI:10.1214/19-STS713.

4 Arkhangelsky D, Imbens GW. Doubly robust identification for causal panel data models. *Econometrics Journal* 2022; **25**. DOI:10.1093/ectj/utac019.
