# Supplementary material for: The impact of elective surgical hubs on elective surgery in acute hospital trusts in England
Source: Nat Commun. 2025 Jul 4;16:6192. doi: 10.1038/s41467-025-60936-6 (PMC12227523; doi:10.1038/s41467-025-60936-6)
Supplement: Supplementary file 2 — Reporting Summary [file 41467_2025_60936_MOESM2_ESM.pdf]

nature portfolio

Corresponding author(s): Geraldine Clarke

Last updated by author(s): Jan 8, 2025

Reporting Summary

Nature Portfolio wishes to improve the reproducibility of the work that we publish. This form provides structure for consistency and transparency in reporting. For further information on Nature Portfolio policies, see our Editorial Policies and the Editorial Policy Checklist.

Statistics

For all statistical analyses, confirm that the following items are present in the figure legend, table legend, main text, or Methods section.

n/a	Confirmed	
☐	☒	The exact sample size (n) for each experimental group/condition, given as a discrete number and unit of measurement
☒	☐	A statement on whether measurements were taken from distinct samples or whether the same sample was measured repeatedly
☐	☒	The statistical test(s) used AND whether they are one- or two-sided<br>*Only common tests should be described solely by name; describe more complex techniques in the Methods section.*
☐	☒	A description of all covariates tested
☐	☒	A description of any assumptions or corrections, such as tests of normality and adjustment for multiple comparisons
☐	☒	A full description of the statistical parameters including central tendency (e.g. means) or other basic estimates (e.g. regression coefficient) AND variation (e.g. standard deviation) or associated estimates of uncertainty (e.g. confidence intervals)
☐	☒	For null hypothesis testing, the test statistic (e.g. F, t, r) with confidence intervals, effect sizes, degrees of freedom and P value noted<br>*Give P values as exact values whenever suitable.*
☒	☐	For Bayesian analysis, information on the choice of priors and Markov chain Monte Carlo settings
☐	☒	For hierarchical and complex designs, identification of the appropriate level for tests and full reporting of outcomes
☐	☒	Estimates of effect sizes (e.g. Cohen's d, Pearson's r), indicating how they were calculated

Our web collection on statistics for biologists contains articles on many of the points above.

Software and code

Policy information about availability of computer code

Data collection	No software was used as analysis used data that had been already collected by third party providers.
Data analysis	Analyses were performed using the gsynth package (version 1.0.9, https://cran.r-project.org/web/packages/gsynth/index.html) in R (version 4.0.2). Code for our analysis is available on GitHub at https://github.com/HFAnalyticsLab/elective_hubs.

For manuscripts utilizing custom algorithms or software that are central to the research but not yet described in published literature, software must be made available to editors and reviewers. We strongly encourage code deposition in a community repository (e.g. GitHub). See the Nature Portfolio guidelines for submitting code & software for further information.

Data

Policy information about availability of data

All manuscripts must include a data availability statement. This statement should provide the following information, where applicable:

- Accession codes, unique identifiers, or web links for publicly available datasets
- A description of any restrictions on data availability
- For clinical datasets or third party data, please ensure that the statement adheres to our policy

This work uses data provided by patients as part of their care and support. Individual patient-level data and data supplied under specific data sharing agreements cannot be made available by the study team. The data were collated, maintained, and quality assured by NHS Digital, now part of NHS England. Requests for access to these data should be directed to the Data Access Request Service, which is part of NHS England (https://digital.nhs.uk/services/data-access-request-service-dars).

Research involving human participants, their data, or biological material

Policy information about studies with human participants or human data. See also policy information about sex, gender (identity/presentation), and sexual orientation and race, ethnicity and racism.

Reporting on sex and gender	The term 'gender' is used to describe how effect estimates were adjusted to control for changes over time in the population-at-risk characteristics as our analysis as gender, not sex, was considered in the study design.<br><br>Effect estimates were adjusted to control for changes over time in the population-at-risk characteristics. For elective activity rates, the population-at-risk is the trust catchment population and we adjusted for the proportion of those who were male, with White ethnicity, and aged 65 years or older. Demographic characteristics of the trust catchment population including the proportions aged 65 years and older, male and with White ethnicity were obtained from the Office for Health Improvement and Disparities' NHS Acute (Hospital) Trust Catchment Populations dashboard.<br><br>For day-case proportion and inpatient length of stay, the population-at-risk is those undergoing surgery and we adjusted for the proportion of those who were male, with White ethnicity, aged 65 years or older, living in the 20% of most deprived areas and with 2 or more Elixhauser comorbidities based on their admissions in the previous 24 months. Characteristics of patients undergoing elective surgery (age, gender, deprivation and number of Elixhauser comorbidities over the preceding 24 months) were obtained for each trust for each month from the Hospital Episode Statistics (HES) dataset. HES is a curated data product containing details about admissions, outpatient appointments and historical accident and emergency attendances at NHS hospitals in England.<br><br>Findings were not disaggregated by gender or any other patient demographic as changes were assessed at the level of Trusts rather than for individual patient groups.<br><br>Table 1 shows the median and inter-quartile range of the population who are from a White ethnic background, who are male, who are aged 65+ years old and the catchment population size at each hub trust type.
Reporting on race, ethnicity, or other socially relevant groupings	See answer on reporting on sex and gender.
Population characteristics	See answer on reporting on sex and gender.
Recruitment	All procedures for elective surgeries during our study period in our included trusts were part of the study population, except for procedures with admission dates outside study period or procedures with admission dates during lockdown (April 2021 – March 2022 exclusive). We also excluded trusts with data missing in one or more months, or those with known organisation changes likely to affect data (e.g., trust mergers). We also excluded trusts specialising in a single surgical area due to less generalisable activity. This could lead to residual unobserved confounding that could bias our estimates.
Ethics oversight	No ethics oversight was required for this study. Written informed consent or participant compensation was not required for this study as it utilised secondary data that had been previously collected and anonymised.

Note that full information on the approval of the study protocol must also be provided in the manuscript.

Field-specific reporting

Please select the one below that is the best fit for your research. If you are not sure, read the appropriate sections before making your selection.

☐ Life sciences ☒ Behavioural & social sciences ☐ Ecological, evolutionary & environmental sciences

For a reference copy of the document with all sections, see nature.com/documents/nr-reporting-summary-flat.pdf

Behavioural & social sciences study design

All studies must disclose on these points even when the disclosure is negative.

Study description	This was a quantitative study using a generalised synthetic control methodology to evaluate the impact of elective surgical hubs on elective surgery in acute hospital trusts in England.
Research sample	This study involved human research participants.<br><br>It used an existing dataset, the Hospital Episode Statistics (HES) dataset. HES is a curated data product containing details about admissions, outpatient appointments and historical accident and emergency attendances at NHS hospitals in England. The data were collated, maintained, and quality assured by NHS Digital, now part of NHS England.<br><br>This study sample was chosen because HES is the main source of data on admissions at NHS Hospitals in England. It includes detailed records of all inpatient admissions across NHS hospitals in England. HES is representative of hospital activity within NHS hospitals in England.

	We included all episodes for patients aged 17 years or over between April 2018 and December 2022 to allow for both pre- and post-intervention data. Each episode represents the time spent by one patient at a single hospital under a single consultant. We linked all episodes within the same hospital admission to generate spells. We included spells with an episode assigned an Office of Population Censuses and Surveys version 4 (OPCS4) code representing an 'intermediate' surgery, as defined by Abbott et al. This includes procedures routinely undertaken in an operating theatre and/or under anaesthesia.<br><br>The covariate-relevant population characteristics of the human research participants were:<br>- Population from a White ethnic background (%), median (IQR): New-hub trusts: 85 (72, 92). Established -hub trusts: 89 (79, 93). Non-hub trusts: 93 (87, 96)<br>- Population who are male (%), median (IQR): New-hub trusts: 48 (48, 50). Established-hub trusts: 50 (48, 51). Non-hub trusts: 48 (48, 50)<br>- Population who are aged 65+ years old (%), median (IQR): New-hub trusts: 19 (14, 21). Established-hub trusts: 19 (16, 21). Non-hub trusts: 20 (18, 23)
Sampling strategy	National study including data from all available hospital records (> 14M procedures) from included trusts in the relevant time period. No sample size calculations were performed. It was determined that the HES dataset was sufficiently large to provide robust and reliable insights for the analysis, given its inclusion of data from all NHS hospitals in England.
Data collection	Data was curated and shared by NHS England, see https://digital.nhs.uk/data-and-information/data-tools-and-services/data-services/hospital-episode-statistics for more detail.<br>HES is collected from Patient Administration Systems at NHS trusts, coded using ICD-10 and OPCS-4, and validated before being stored in national data warehouses managed by NHS Digital. The researchers were not present during the data collection procedure. As this study only used existing datasets, blinding was not applicable.
Timing	Our study period was April 2018 – December 2022.
Data exclusions	Some trusts and procedures were excluded from the study. We summarise these Supplementary Figure 1. Flowchart of inclusions and exclusions. Exclusions were done for the following reasons:<br>169 trust codes without available data on catchment population (this includes many independent sector providers in HES)<br>23 trusts excluded for issues with data quality and comparability with other trusts:<br>- Merged with other trusts during the study period (no comparable historic data)<br>- Single-specialty trusts (cancer, children's, women's trusts, ophthalmology-only)<br>- Hub trusts where start date of hub was not known<br>- Trusts with recorded data quality issues (e.g. from HES data quality reports)<br><br>141,017 procedures with admission dates outside study period (April 2018 – December 2022)<br>1,269,727 procedures with admission dates during lockdown (April 2021 – March 2022 exclusive)<br>Exclusion criteria were pre-established but an element of judgment was required to decide if trusts had data quality issues sufficient for exclusion.
Non-participation	No participants dropped out as by definition patients needed to have completed an elective surgery to be included.
Randomization	Participants were not allocated to random groups. Participant allocation was not relevant to this study as the study was observational and did not allocate participants into experimental groups. The covariates of proportions aged 65 years and older, male and with White ethnicity were included in the model to control for confounding. In addition, the generalised synthetic control method used for this analysis accounts for the likely presence of unmeasured confounders and of effects that vary across time and trusts.

Reporting for specific materials, systems and methods

We require information from authors about some types of materials, experimental systems and methods used in many studies. Here, indicate whether each material, system or method listed is relevant to your study. If you are not sure if a list item applies to your research, read the appropriate section before selecting a response.

Materials & experimental systems

n/a	Involved in the study
☒	☐ Antibodies
☒	☐ Eukaryotic cell lines
☒	☐ Palaeontology and archaeology
☒	☐ Animals and other organisms
☒	☐ Clinical data
☒	☐ Dual use research of concern
☒	☐ Plants

Methods

n/a	Involved in the study
☒	☐ ChIP-seq
☒	☐ Flow cytometry
☒	☐ MRI-based neuroimaging

Plants

Seed stocks

Report on the source of all seed stocks or other plant material used. If applicable, state the seed stock centre and catalogue number. If plant specimens were collected from the field, describe the collection location, date and sampling procedures.

Novel plant genotypes

Describe the methods by which all novel plant genotypes were produced. This includes those generated by transgenic approaches, gene editing, chemical/radiation-based mutagenesis and hybridization. For transgenic lines, describe the transformation method, the number of independent lines analyzed and the generation upon which experiments were performed. For gene-edited lines, describe the editor used, the endogenous sequence targeted for editing, the targeting guide RNA sequence (if applicable) and how the editor was applied.

Authentication

Describe any authentication procedures for each seed stock used or novel genotype generated. Describe any experiments used to assess the effect of a mutation and, where applicable, how potential secondary effects (e.g. second site T-DNA insertions, mosiacism, off-target gene editing) were examined.
