# Supplementary material for: The impact of elective surgical hubs on elective surgery in acute hospital trusts in England
Source: Nat Commun. 2025 Jul 4;16:6192. doi: 10.1038/s41467-025-60936-6 (PMC12227523; doi:10.1038/s41467-025-60936-6)
Supplement: Supplementary file 3 — Transparent Peer Review file [file 41467_2025_60936_MOESM3_ESM.pdf]

nature portfolio

Peer Review File

The impact of elective surgical hubs on elective surgery in acute hospital trusts in England

Corresponding Author: Dr Geraldine Clarke

This file contains all reviewer reports in order by version, followed by all author rebuttals in order by version.

Version 0:

Reviewer comments:

Reviewer #1

(Remarks to the Author)
Summary

This paper evaluates the impact of opening surgical hubs - ringfenced centres for surgical activities within hospital trusts in the English NHS - on a number of relevant indicators capturing the volume and efficiency in service provision. The study spans the COVID-19 lockdown period and employs generalized synthetic controls for causal impact evaluation. The authors report significant effects on total activities, particularly on high-volume, low-complexity (HVLC) activities targeted by the hubs. They also found reductions in length of stay, although this was not statistically significant for hub-targeted activities. Overall, the paper presents a carefully performed policy evaluation within the context of the English NHS. The authors have clearly put considerable effort into their analysis and present findings that could have important implications for healthcare policy. However, several improvements could enhance its general applicability and can make the applied methodology more transparent.

Theoretical Framework and Hypotheses

The paper would benefit from a clearer outline of the story the authors are telling. What are the mechanisms by which they expect these ringfenced hubs within hospital trusts to affect the provision of care, and ultimately, patient outcomes? This discussion should also consider potential drawbacks or unintended consequences. For instance, is more activity always better, or could there be a risk of supplier-induced demand to justify the existence of the hubs?
The introduction should present more explicit hypotheses being tested, which would help frame the subsequent analysis and make it easier for readers to follow the logical flow of the paper. Some of this discussion appears in the later sections of the paper and could be beneficially moved to the introduction to set the stage for the analysis that follows.

To enhance the broader applicability of these findings beyond England, it would be valuable to see how this work relates to international literature on similar health policy interventions. Is there precedent for such surgical hubs in other healthcare systems, and if there are evaluations available, how do the findings of this study compare?

Methodological Clarity

The methodological approach, using generalized synthetic controls, is sophisticated and appropriate for this type of policy evaluation. However, more explanation would be beneficial for readers who may not be familiar with this method. In particular, the paper would benefit from a more in-depth discussion of the assumptions behind the generalized synthetic control estimator, and whether they are likely to be met in the context of this study? On line 289 the authors say that the assumption is that the outcome trends for hub-trusts would be the same as the synthetic control. This is somewhat hard to interpret as an assumption as the synthetic control is already the result of the estimation process.

It would be helpful to clarify how this approach can estimate treatment effects for each treated unit separately, as opposed to for example the differences-in-differences approach, which considers average treatment effects only.) Additionally, more explanation is needed regarding the confidence intervals around the individual treatment effects, presented in

Supplementary Figure . Are these related to the bootstrap procedure mentioned?

More elaboration is needed on the selection mechanism for trusts opening hubs. Do we have any descriptive information on trust characteristics, beyond the patient case mix presented in Table 1? The authors mention that these hubs may have had more resources and that other initiatives targeting efficiency improvements may have been ongoing. This raises questions about the policy implications – are we truly observing the effects of opening hubs, or are we seeing the results of a number of reforms in a subset of hospital trusts?

Related to the above, The COVID-19 pandemic appears to have been a key catalyst for opening new hubs. This raises important questions about the ability to separate the effects of opening new hubs from trusts' reactions to the pandemic. What might be reasons why some trusts would choose not to open hubs? These contextual factors may help interpreting the results and understanding their generalizability.

Further considerations (minor)

Given the multiple analyses and hypotheses being tested, it would be good to hear the authors' thoughts on multiple hypothesis correction. This is an important consideration in complex policy evaluations like this one and addressing it explicitly would strengthen the robustness of the findings.

While the current analysis provides valuable insights, it might be worthwhile to consider additional measures of trust performance such as patient waiting times, readmission rates, and 30-day hospital mortality. These could provide a more comprehensive picture of the hubs' impact on overall healthcare quality and efficiency.

It would also be interesting to see the effect on elective non-HVLC activities to differentiate impacts. This could help isolate the specific effects of the hubs on their targeted activities versus broader impacts on the trusts' operations.

Terminology and Accessibility

A significant challenge for the paper's broader impact is its use of language specific to the NHS. To make the work more accessible to an international audience, I would suggest using more generally known terms or, at the very least, clearly defining NHS-specific terminology. For example, terms like "elective activity" (which might be misunderstood as optional rather than non-emergency), "consultant," "trust," and "ringfencing" need clear explanations for readers unfamiliar with the NHS structure.

Similarly, technical terms such as "day case proportion" or "elective activity rate" are only explained later in the study design section. Making the introduction and discussion less technical and more accessible to readers outside the context of the English NHS would significantly enhance the paper's impact.

Data Visualization and Presentation (minor)

A small suggestion to Supplementary Figure 2: it could be more informative if it showed "New" and "Established trusts" (and ideally the "Non-hub" trusts) on the same graphic, with different colours.

Conclusion
This paper presents a valuable contribution to our understanding of the impact of surgical hubs within the NHS. With some revisions to enhance its accessibility, strengthen its methodological explanations, and broaden its context, it could provide important insights for healthcare policy both within the UK and internationally. Addressing these points would significantly enhance the paper's suitability for publication in Nature Communications.

(Remarks on code availability)

Reviewer #2

(Remarks to the Author)
Major Comments:
---

* This is important and timely work, as the problems which the GIRFT policy aimed to solve are clearly of national importance. Whether the framing and narrative of the paper is sufficiently broad -- i.e. of international importance -- for a general readership has to be left to the discretion of the editor. However, more could likely be done to re-frame the paper to give its international relevance more salience. Despite this, I do think this research design, i.e. the use of administrative data like HES -- and despite the problems mentioned below -- is a progressive way to tackle temporally pertinent healthcare

issues.

* This paper initially seems to be suffering from a dearth of academic references. This may seem like a harsh major comment to give, but it is symptomatic of the lack of good academic style in general. Yes, there are papers listed in the bibliography, but they are often not referenced inline in the main body of the article. A lot of work would be required to rewrite this into an academic article, away from what is currently more of a white paper/policy brief.

* Why generalised synthetic controls? Why are they appropriate here? Perhaps most importantly; what are the treated units?! What is the estimand of interest? A lot of key information about this research design is lacking prominence/careful delivery (and accurate technical exposition). When parts of this important information does arrive in the Discussion, it is unfortunately a little too late, and a little too superficial.

* Unfortunately, a major geographical component is missing from this analysis. Not only is the NHS an increasingly and somewhat intensely regional institution, the regional variations have enormous affect on the demand for services which the NHS supplies, not least the type of services supplied as discussed in this paper. When this does happen -- discussion in terms of elective activity rate -- this is again unfortunately far too light.

* Perhaps most unfortunately, there is absolutely no way a willing replicator of the work could even begin to try to reproduce these results mentioned within; far too much detail is lacking, and details are covered with insufficient depth.

Minor:
---

1. There are _a lot_ of keywords! Is this in keeping with the journal's guide for authors?
2. The first paragraph of the results section would likely better live in the 'Data and Methods' section, where it could be further bolstered. Supplementary Figure 1 goes someway towards this, but a lot more visualisation could be done here to help the reader better understand the dataset(s) and the properties of the estimator's inputs..
3. The figures generally leave a little to be desired, both in their production and in their resolution; more work can be done to improve their usefulness, and they can be expanded substantially as expressed above.

(Remarks on code availability)
Not only are there no replication materials available, as mentioned in my review, it would be almost infinitely impossible to even begin to attempt to replciate this study.

Version 1:

Reviewer comments:

Reviewer #1

(Remarks to the Author)
The authors have carefully addressed all my comments and implemented many of my suggestions. I think the paper is now in publishable shape.

(Remarks on code availability)

Reviewer #2

(Remarks to the Author)
Major comments:

I was glad to receive the chance to again review what is clearly a much improved version of this paper. It certainly addresses some of the earlier points raised but, however, it leaves others relatively insufficiently complete.

Primarily, I feel like the referencing is now much more in-line with what would be expected with a journal like Nature Communications. While the subject and writing style matter may or may not be more like a white-paper or specifically that more like one expected from a leading Public Health journal, it is much improved. I commend the authors for this. It feels much more like an academic article than it did previously.

Specifically, it much better referenced, and the broader problem statement is more adequately introduced and defined in the opening paragraphs (and it better motivates the need for the study).

It also addresses a previous concern about the clear definition of treated and control units.

However, a major concern remains regarding the enunciation of the methods. There is almost certainly no way -- as the paper is currently written -- to understand what the GSC method is actually doing. I am of the general opinion that in applied papers, there is no need to formally re-derive a method., or even advance it. However, in this case, the technical detail on exactly what it is doing is so lacking that it makes it hard for a reader outside of the (Generalised) Synthetic Control literature

to actually understand what this method does. There are a large number of questions, and equations are necessary to further elaborate things such as 'How are latent factors and loadings generated?', 'How are counterfactual outcomes predicted?', 'How are treatment effects calculated?'. These are not mathematically intractable things to express, and I feel like if they come _after_ the discussion and conclusion of the papers as is common in journals such as this, they would be 'optional things for the reader to consider if and as appropriate' and provide great value.

That is to say, again: please consider bringing Appendix 3 into the main text!

I would also raise the fact that HES data represents one specific type of (secondary) care; more should be done to assuage readers about the suitability of this data for this problem. There are, for example, many reasons why HES is not more routinely used in academic research (which is an altogether separate shame), in that it forms just one piece of the (healthcare demand) side of the puzzle (i.e. it omits primary care).

Further points were raised by both reviewers on the quality of the figures. The new 'Figure 1' is relatively useful, but can likely be much more adequately designed to inform the reader of the 'flow' of the data within the NHS and, accordingly, through the paper (and GSC algorithm). Figma is the relevant tool of choice at this point in time.

Minor comments:

"12 national" --> "twelve national" and "3 hospitals" --> "three hospitals", please! Integers less than twenty one should be words, and integers above 21 should be in numbers (at least outside of the reporting of estimated effects). It may be that the editorial team is happy to take care of things like this after the fact.

While the second through figures are useful, they -- like Figure 1 -- should also be lossless (.pdf, .svg) as appropriate.

(Remarks on code availability)
The code looks relatively complete, well written, and well documented. However, it is not a complete replication package due to constraints in data availability (by license).

Open Access This Peer Review File is licensed under a Creative Commons Attribution 4.0 International License, which permits use, sharing, adaptation, distribution and reproduction in any medium or format, as long as you give appropriate credit to the original author(s) and the source, provide a link to the Creative Commons license, and indicate if changes were made.
In cases where reviewers are anonymous, credit should be given to 'Anonymous Referee' and the source.
The images or other third party material in this Peer Review File are included in the article's Creative Commons license, unless indicated otherwise in a credit line to the material. If material is not included in the article's Creative Commons license and your intended use is not permitted by statutory regulation or exceeds the permitted use, you will need to obtain permission directly from the copyright holder.
To view a copy of this license, visit https://creativecommons.org/licenses/by/4.0/

Reviewer #1

Note: Paragraph numbers noting changes are based on the untracked version of the manuscript.

1. **Summary.**

    a. **This paper evaluates the impact of opening surgical hubs - ringfenced centres for surgical activities within hospital trusts in the English NHS - on a number of relevant indicators capturing the volume and efficiency in service provision. The study spans the COVID-19 lockdown period and employs generalized synthetic controls for causal impact evaluation. The authors report significant effects on total activities, particularly on high-volume, low-complexity (HVLC) activities targeted by the hubs. They also found reductions in length of stay, although this was not statistically significant for hub-targeted activities.**
    Overall, the paper presents a carefully performed policy evaluation within the context of the English NHS. The authors have clearly put considerable effort into their analysis and present findings that could have important implications for healthcare policy. However, several improvements could enhance its general applicability and can make the applied methodology more transparent.

2. **Theoretical Framework and Hypotheses**

    a. **The paper would benefit from a clearer outline of the story the authors are telling. What are the mechanisms by which they expect these ringfenced hubs within hospital trusts to affect the provision of care, and ultimately, patient outcomes?**

    Thank you for the comment. Ahead of our analysis, we developed a logic model for the GIRFT elective hubs national programme to understand the outputs and outcomes related to the programme. This informed the outcome measures for our analysis. This is now included in the manuscript (Figure 1):

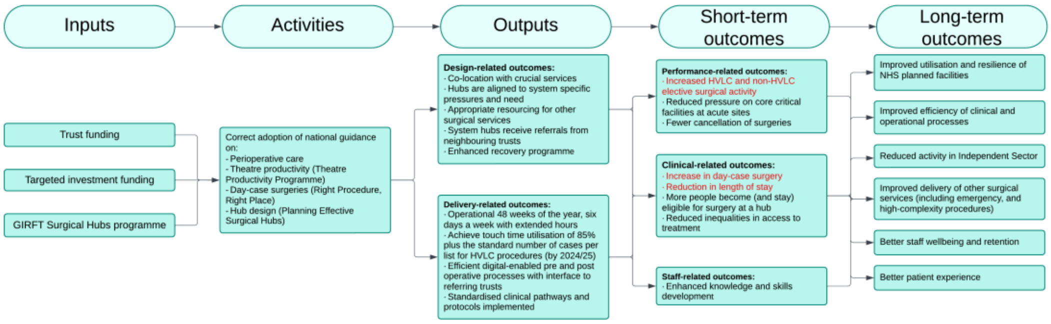

    To better walk the reader through the story we are telling, we have not extensively rewritten the introduction. After introduction paragraphs 1-4 which

reflect on the context, organisation and investment in hubs, we introduce the logic model and expected outcomes:

> Introduction paragraph 5*: "The GIRFT HVLC programme aims to enable hubs to achieve several projected short-term outcomes including performance related improvements such as increased activity and fewer cancellations; clinical benefits, such as more day-case surgeries, shorter post-surgery stays and reduced inequalities in access to treatment; and staff-focused benefits such as enhanced skills and knowledge development, predictable working patterns, and better wellbeing and retention (Figure 1)[19]. Ultimately these changes are expected to result in better longer-term utilisation and resilience of NHS planned facilities and resources, along with greater efficiency."*

Then we discuss the existing evidence on hubs:

> Introduction paragraph 6: *"Evidence on the impact of hubs, or their international equivalents, on potential outcomes is heterogenous and limited. A rapid review of 12 national and international studies published between 2015 and 2022 found evidence to suggest that hubs can improve clinical outcomes such as length of hospital stay, operative and post-operative complications, readmission rates and surgery quality, and found limited evidence of hubs on performance outcomes such as efficiency, utilisation, volume, cancellations, and time from diagnosis to treatment[20]. Other studies have reported reduced duration of stay after surgery[11–13,15,21,22]. A study of 3 hospitals in Norway suggested ringfencing can improve hospital efficiency[14]. A study in Canada found that continuity of care and patient satisfaction could be maintained when introducing additional ring-fenced capacity at a single site[10]. However, most studies were focused on a single location with considerable variation in the types of hubs and surgical specialties analysed leading to a lack of generalisability. Additionally, they often used weak methods like before-and-after designs, which can lead to incorrect conclusions about effects. Only one study attempted to examine the causal impact of hubs[14]."*

Next, we bring these factors together to provide motivation for the evaluation

> Introduction paragraph 7*: "The increasing number of hubs and investment in new sites warrants robust causal evidence of their impact on key outcomes. The national rollout provides the opportunity to assess causal impacts across the wider health care system by comparing trusts with hubs to those without. Here, we perform a causal evaluation of the impact of hubs on system-wide elective care performance (elective surgery rate) and clinical (inpatient length of hospital stay and day-case proportion) outcomes nationally using the generalised synthetic control method (GSC)[23]. The GSC approach is suitable for evaluating the impact of a health care intervention (in this case having a hub) against a counterfactual (if the hub did not exist)*

where there are multiple treated units, staggered intervention start dates and data is available at multiple time points. This modelling framework effectively accounts for unique aspects of typical health care data, namely, the likely presence of unmeasured confounders and of effects that vary across time and trusts[24,25].”

We thank the reviewer for this comment and believe that the Introduction is much improved with these changes.

b. **This discussion should also consider potential drawbacks or unintended consequences. For instance, is more activity always better, or could there be a risk of supplier-induced demand to justify the existence of the hubs?**

We have now substantially rewritten the last paragraph of the discussion to include more on unintended consequences:

> Discussion paragraph 9: “*Further research is required to understand other impacts of elective surgical hubs (Figure 1), such as the effects on staff-related outcomes and inequalities in access to treatment, as well as any unintended consequences. This includes examining quality and safety of care for patients in hubs, such as whether readmission or mortality rates have changed, as well as understanding if there are unintended consequences on outcomes for patients treated outside of hubs, such as those with higher-complexity surgeries not offered in hubs*.”

While we did not evaluate this, a major potential unintended consequence that we have mentioned is the effect on emergency care and acute care outside hubs, such as patients needing higher-complexity surgeries. In this case, we believe these issues are likely more of concern than supplier-induced demand, as hubs are delivering high-volume surgeries within a system with an existing backlog of people waiting for care. Our analyses also examine elective surgeries at a trust level, so encompasses increases in activity performed outside hubs as well.

c. **The introduction should present more explicit hypotheses being tested, which would help frame the subsequent analysis and make it easier for readers to follow the logical flow of the paper. Some of this discussion appears in the later sections of the paper and could be beneficially moved to the introduction to set the stage for the analysis that follows.**

The Introduction has now been extensively rewritten to help the flow of the paper. See also response to Reviewer 1 2a above. In particular, we have now included further information on the specific hypotheses being tested:

> Introduction paragraph 7*: “…Here, we perform a causal evaluation of the impact of hubs on system-wide elective care performance (elective surgery rate) and clinical (inpatient length of hospital stay and day-case proportion) outcomes nationally using the generalised synthetic control method (GSC)[23]…”*

Introduction paragraph 8*: "In our analysis we distinguish between 'established' hubs that opened long before the COVID-19 pandemic (operational before 2019) and 'new' hubs that opened just before, or in response to, the pandemic (operational from 2019 onwards). Trusts with established hubs (established-hub trusts) likely had set procedures in place before the start of the pandemic in March 2020, potentially positioning them better than other trusts to recover planned surgeries lost during the government-mandated COVID-19 lockdown. In contrast, trusts with new hubs (new-hub trusts), many of which opened during lockdown typically in response to the suspension of planned services during the lockdown, may have taken time to establish their operational standards after the lockdown. We examined impacts on all (or total) elective surgery and, assuming hubs focus on HVLC elective surgery, on the subset of HVLC elective surgery to distinguish the contribution of hubs to the observed trust-wide effects."*

The inclusion of the logic model (Figure 1) will help readers to understand the overall theory of change for elective hubs, and then the aspect of this that we are looking at. This make clears there are other outputs and outcomes of hubs which we have not been able to evaluate. These are further referenced in Discussion (paragraph 9) – see response to Reviewer 1 2b.

d. **To enhance the broader applicability of these findings beyond England, it would be valuable to see how this work relates to international literature on similar health policy interventions. Is there precedent for such surgical hubs in other healthcare systems, and if there are evaluations available, how do the findings of this study compare?**

We have now added further details and reference to international (and UK/England) literature on surgical hubs in the Introduction:

Introduction paragraph 2*: "Similar ringfencing approaches have been used across the UK and other countries including Ireland, Norway, Canada and Australia*[10–15]*."*

Introduction paragraph 6: *"Evidence on the impact of hubs, or their international equivalents, on potential outcomes is heterogenous and limited. A rapid review of 12 national and international studies published between 2015 and 2022 found evidence to suggest that hubs can improve clinical outcomes such as length of hospital stay, operative and post-operative complications, readmission rates and surgery quality, and found limited evidence of hubs on performance outcomes such as efficiency, utilisation, volume, cancellations, and time from diagnosis to treatment*[20]*. Other studies have reported reduced duration of stay after surgery*[11–13,15,21,22]*. A study of 3 hospitals in Norway suggested ringfencing can improve hospital efficiency*[14]*. A study in Canada found that continuity of care and patient satisfaction could be maintained when introducing additional ring-fenced capacity at a single site*[10]*."*

While surgical hubs are not new and have been studied in other healthcare systems, what we have found is that most studies focus on individual ring-fenced sites (rather than studying system-wide effects of this change in healthcare design and delivery). This makes it difficult for us to draw direct comparisons to our current work. There is also a lot of variation in the purpose of ring-fencing. For example, during the pandemic it was often implemented as a means of infection control, with different outcomes of interest. See also response to Reviewer 2 1a.

3. **Methodological Clarity**

   a. **The methodological approach, using generalized synthetic controls, is sophisticated and appropriate for this type of policy evaluation. However, more explanation would be beneficial for readers who may not be familiar with this method. In particular, the paper would benefit from a more in-depth discussion of the assumptions behind the generalized synthetic control estimator, and whether they are likely to be met in the context of this study?**

   We have included additional information on method suitability in the Introduction and Methods sections:

   > Introduction paragraph 7: *"The GSC approach is suitable for evaluating the impact of a health care intervention (in this case having a hub) against a counterfactual (if the hub did not exist) where there are multiple treated units, staggered intervention start dates and data is available at multiple time points. This modelling framework effectively accounts for unique aspects of typical health care data, namely, the likely presence of unmeasured confounders and of effects that vary across time and trusts[24,25]."*
   >
   > Methods, Statistical Analysis*: "GSC offers flexibility for causal inference in our context for the following reasons:*
   >
   > - *It accounts for unique aspects of typical health care data, namely, the likely presence of unmeasured confounders and of effects that vary across time and trusts. For example, in our data this might include differences between trusts in relation to why they choose to open a hub or not, or other time-varying differences between trusts that could affect outcomes.*
   > - *It allows for multiple treated units, enhancing the power to detect effects and allowing mitigating the impact of any potential idiosyncrasies that a few hub trusts may show compared to others.*
   > - *It supports varying treatment periods among treated units, which is useful for incorporating staggered hub opening dates in the new-hub trusts analysis.*
   > - *It conveniently incorporates observed time-varying covariates by including them as main effects in the model.*

- *It provides coherent measures of accuracy, such as standard errors and confidence intervals for effect estimates, using a parametric bootstrap procedure."*

More information on the rationale for choosing the generalised synthetic control method, as well as technical details of the method, are also now included in the Supplementary Material (Appendix 3). See also response to Reviewer 2 1c.

b. **On line 289 the authors say that the assumption is that the outcome trends for hub-trusts would be the same as the synthetic control. This is somewhat hard to interpret as an assumption as the synthetic control is already the result of the estimation process.**

Thank you for pointing this out, this was an error. We have now rewritten the description of the GSC method in Methods section:

> Statistical Analysis*: "GSC uses interactive fixed effect regression modelling and synthetic control methodology to estimate the outcome changes in hub-trusts relative to a synthetic control, or counterfactual, which estimates the outcomes trends that would have been seen without the hub. Synthetic controls are constructed using data from non-hub trusts across all time points and data from hub-trusts in the pre-intervention period only."*

We have also added substantial technical details in the Supplementary Material Appendix 3 for the interested reader.

c. **It would be helpful to clarify how this approach can estimate treatment effects for each treated unit separately, as opposed to for example the differences-in-differences approach, which considers average treatment effects only.)**

Supplementary Appendix 3 has been updated to include a detailed presentation of the GSC method. This includes the linear factor structure and the steps to estimate individual treatment effects for each treated unit at each treated time period. This also includes details of how these individual effect estimates are aggregated across treated units to estimate an average treatment effect at each post-intervention time period.

d. **Additionally, more explanation is needed regarding the confidence intervals around the individual treatment effects, presented in Supplementary Figure. Are these related to the bootstrap procedure mentioned?**

Full details of the parametric bootstrap procedures used to obtain uncertainty estimates of the GSC estimator in the original research article by Xu et al. We have explicitly noted this in our description of the GSC method in Supplementary Appendix 3. We have also added a note to specify how the confidence intervals for the individual treatment effects were derived in the legend for Supplementary Figures 9 and 10.

e. **More elaboration is needed on the selection mechanism for trusts opening hubs.**

We have included a new paragraph in the Introduction (paragraph 4) which sets out some of the factors related to a trust decision to set up the hub.

> Introduction paragraph 4: "*The decision to set up an elective surgical hub can depend on several factors. These include funding and resources. Trusts that bid for TIF funding were required to develop detailed proposals outlining needs, benefits and potential outcomes as well as comprehensive financial plans. Better financed trusts, or those successful in bidding for funds, may be more likely to set up a hub, or be running additional initiatives targeting efficiency improvements. Additionally, trusts with higher patient demand or waiting lists, or those with a history of successful elective surgery programs, may prioritise the creation of a hub. Finally, different trusts may have different strategic priorities, management structures and operational capacity all of which may play a role in the decision to set up a hub and other initiatives. Against this background, COVID-19 was also a catalyst for trusts to open a hub – enabling clearly demarcated areas, one for acute activity including COVID-19 patients and one completely separate area with ringfenced staff and theatres for planned elective activity."*

We have also updated the limitations in the Discussion to reflect on this point specifically and how it could lead to confounding and selection biases.

> Discussion paragraph 8: "..*Thirdly, we were unable to account for systemic differences between trusts, such as their decision to set up a hub or other broader investments in elective care that may go alongside that. These systemic differences could confound the relationship between hubs and improved trust-wide outcomes. For example, broader investments in elective care may influence whether trusts open a hub and may also separately be associated with increased elective surgical activity outside the hub. There may also be selection bias if the hub trusts are not representative of trusts without hubs…"*

See also response to Reviewer 1 3.g.

f. **Do we have any descriptive information on trust characteristics, beyond the patient case mix presented in Table 1?**

We have now referenced our previous study (Tracey et al. reference 16) which examined and compared the characteristics of NHS acute trusts with hubs and NHS acute trusts nationally in the Introduction:

> Introduction paragraph 3: *"…The distribution of hubs across the country differs widely by configuration, capacity, care provision and contextual setting within acute hospital trusts[16]."*

We also refer to this in the Results:

> Results paragraph 1: "*An earlier mapping study of hubs in England found that they are widespread across NHS regions in England, mainly in more densely populated areas, with significant variation in the hub types, specialties, care provision, and their healthcare provider contexts[16].*"

Tracey et al. was based on surveys sent out to the trusts evaluated in this study and found that hubs were widespread across NHS regions, with significant variation in the hub types, specialties, care provision and healthcare provider contexts.

g. The authors mention that these hubs may have had more resources and that other initiatives targeting efficiency improvements may have been ongoing. This raises questions about the policy implications – are we truly observing the effects of opening hubs, or are we seeing the results of a number of reforms in a subset of hospital trusts?

Thank you for this comment; this is an important point which we had not explicitly addressed previously. We are unable to determine whether the effects we observe at new-hub trusts reflect increased capacity as a result of additional resources, rather than efficiency due to the hub, although improvements in day-case proportion may suggest improvements in efficiency. This is unlikely to be an issue at established-hub trusts where we can more confidently assume that there was no increase in capacity. We have now added this explicitly as the fourth limitation in the Discussion:

> Discussion paragraph 8: *"…Fourthly, in the new-hub trusts analysis, we couldn't determine whether increased rates reflected greater efficiency from setting up a hub, or simply increased capacity from additional resources. However, the improvement in the day-case proportion across all elective surgeries at new-hub trusts might indicate the former…"*

We are also unable to explicitly account for whether hubs may have had other initiatives targeting efficiency improvements. We have added a new paragraph to the Introduction (see response to Reviewer 1 3e above) setting out the reasons why a trust might decide to set up a hub and speculate that it is likely that trusts who invest in hubs would have also invested in other changes to their elective activity.

However, these other investments (at the time of our evaluation) were not homogeneous or systematic across hub trusts. Some non-hub trusts may also have had investments into elective care that were not hub-related as well. Because we are analysing average effects across multiple trusts, we expect to at least partially account for some of this noise. The generalised synthetic control method also attempts to reduce bias from unmeasured confounders.

h. **Related to the above, The COVID-19 pandemic appears to have been a key catalyst for opening new hubs. This raises important questions about the ability to separate the effects of opening new hubs from trusts' reactions to the pandemic. What might be reasons why some trusts would choose not to open hubs? These contextual factors may help interpreting the results and understanding their generalizability.**

   We agree that this is an important point, and we have included an explicit limitation noting that we are unable to discount the possibility that the pandemic may have affected our results thus limiting the generalisability of our work:

   > Discussion paragraph 8: *"…Finally, the COVID-19 pandemic severely disrupted planned care across all trusts, which had still not fully recovered activity at the time of our evaluation and profoundly affected the health system more broadly than planned surgery alone. Many hubs opened during this time as well, to aid with infection control and attempt to reduce the disruption to services. In our analyses, we accounted for the immediate effects of pausing planned care by removing the government lockdown period and performing sensitivity analyses to understand the effect of modelling different time periods. Although the model accounts for these trends, which were common across control trusts as well as hub trusts, we cannot discount the effect this may still have on our results and their generalisability to future contexts."*

   Trusts likely balanced a number of factors in making a decision to open (or not) a hub, including whether there was available financing, buy-in from senior leaders, available facilities, and time and resources needed to rearrange services. We have included a new paragraph in the Introduction reflecting on the decision to set up a hub (see response to Reviewer 1 3e above).

4. **Further considerations (minor)**

   a. **Given the multiple analyses and hypotheses being tested, it would be good to hear the authors' thoughts on multiple hypothesis correction. This is an important consideration in complex policy evaluations like this one and addressing it explicitly would strengthen the robustness of the findings.**

      We agree that multiple hypothesis testing is an important consideration in this evaluation. We considered this both for our main results reported in Table 3 and for the individual hub estimates reported in Supplementary Figures 9 and 10.

      For our main results we considered applying a Bonferroni correction. For our analysis, this would have meant applying a significance level of 0.004 (0.05 [alpha level] / 12 [total number of tests reported in table 3]). As we had pre-specified the outcomes we would calculate (3 outcomes * 2 activity types * 2 hub types) we considered this to be overly restrictive, increasing the risk of a

Type II error. We have however now added indicators of the size of the p-values to Table 3.

For the individual hub estimates we treated any sporadic statistically significant results as likely type 1 errors and did not interpret these as being meaningfully different in our results or discussion. We have now added a line to the description of the Supplementary Figures 9 and 10 displaying these results to make this explicit.

b. **While the current analysis provides valuable insights, it might be worthwhile to consider additional measures of trust performance such as patient waiting times, readmission rates, and 30-day hospital mortality. These could provide a more comprehensive picture of the hubs' impact on overall healthcare quality and efficiency.**

We agree that waiting times, readmission rates, and mortality are important measures of trust performance. Our analysis aims to focus more on trust-wide changes to elective activity as an indicator of increased output, and we have acknowledged that future research on these is needed to fully evaluate hubs in the last paragraph of the Discussion:

> Discussion paragraph 9: *"…Further research is required to understand other impacts of elective surgical hubs (Figure 1), such as the effects on staff-related outcomes and inequalities in access to treatment, as well as any unintended consequences. This includes examining quality and safety of care for patients in hubs, such as whether readmission or mortality rates have changed,…"*

We have also clearly set out outputs and outcomes in the new logic model (Figure 1. We are also aware of current research led by a separate institution which is investigating these outcomes, and we deemed these additional measures out of scope for our work with this in mind (and particularly as the analysis presented here is already quite complex).

c. **It would also be interesting to see the effect on elective non-HVLC activities to differentiate impacts. This could help isolate the specific effects of the hubs on their targeted activities versus broader impacts on the trusts' operations.**

We chose to examine HVLC elective activity as a proxy measure estimating the type of surgeries specifically targeted by hubs, but we also included analysis of total elective activity, which includes non-HVLC elective activity. The effect of non-HVLC activities is the difference between these. In particular, we found that at new hub trusts, any impact in total surgeries was likely due to the impact of HVLC elective surgeries. We have added a line to mention this in the second paragraph of the Discussion:

> Discussion paragraph 2: "*However, the estimated increase in the rate of total and HVLC elective surgeries at new-hub trusts compared to the synthetic control was similar, suggesting, firstly, that any rise in total surgeries was mainly due to more HVLC surgeries and, secondly,*

that there was no negative impact of opening a hub on non-HVLC elective surgeries in the wider trust."

5. **Terminology and Accessibility**

   a. **A significant challenge for the paper's broader impact is its use of language specific to the NHS. To make the work more accessible to an international audience, I would suggest using more generally known terms or, at the very least, clearly defining NHS-specific terminology. For example, terms like "elective activity" (which might be misunderstood as optional rather than non-emergency), "consultant," "trust," and "ringfencing" need clear explanations for readers unfamiliar with the NHS structure. Similarly, technical terms such as "day case proportion" or "elective activity rate" are only explained later in the study design section. Making the introduction and discussion less technical and more accessible to readers outside the context of the English NHS would significantly enhance the paper's impact**

   We have now changed and defined NHS-specific terminology language. While we've decided to continue using the word 'elective' as this is used in other country contexts, we are defining this at the beginning of the Introduction as planned care. We have changed 'activity' to 'surgery', which we think is both clearer and more precise. We decided to keep 'trust' as other terms (e.g. provider) would still need to be defined, but we are defining this in our introduction. These changes are implemented throughout.

6. **Data Visualization and Presentation (minor)**

   a. **A small suggestion to Supplementary Figure 2: it could be more informative if it showed "New" and "Established trusts" (and ideally the "Non-hub" trusts) on the same graphic, with different colours.**

   Thank you for this suggestion. Supplementary 2 has been replaced with a new figure using different colours for the opening of new- and established-hub trusts. After January 2019, all trusts which opened their first new hub would have been considered new hub trusts, so the bottom of the stacked bar remains stable over time after that time point. Non-hub trusts are not included in this graphic because they never opened hubs (within our study trusts this number would have remained consistent over time).

7. **Conclusion**

   a. **This paper presents a valuable contribution to our understanding of the impact of surgical hubs within the NHS. With some revisions to enhance its accessibility, strengthen its methodological explanations, and broaden its context, it could provide important insights for healthcare policy both within the UK and internationally. Addressing these points would significantly enhance the paper's suitability for publication in Nature Communications.**

   Thanks for your comments.

Reviewer #2 (Remarks to the Author):

1. **Major Comments**

    a. **This is important and timely work, as the problems which the GIRFT policy aimed to solve are clearly of national importance. Whether the framing and narrative of the paper is sufficiently broad -- i.e. of international importance -- for a general readership has to be left to the discretion of the editor. However, more could likely be done to re-frame the paper to give its international relevance more salience. Despite this, I do think this research design, i.e. the use of administrative data like HES -- and despite the problems mentioned below -- is a progressive way to tackle temporally pertinent healthcare issues.**

    Thank you for your comments. The introduction and discussion have now been extensively updated. To ease understanding for an international readership, we have changed or explained the England-specific terminology.

    To broaden interest and relevance for an international readership we have included additional information and discussion in relation to international literature in the Introduction and Discussion:

    > Introduction paragraph 2*: "Similar ringfencing approaches have been used across the UK and other countries including Ireland, Norway, Canada and Australia[10–15]."*
    >
    > Introduction paragraph 6: *"Evidence on the impact of hubs, or their international equivalents, on potential outcomes is heterogenous and limited. A rapid review of 12 national and international studies published between 2015 and 2022 found evidence to suggest that hubs can improve clinical outcomes such as length of hospital stay, operative and post-operative complications, readmission rates and surgery quality, and found limited evidence of hubs on performance outcomes such as efficiency, utilisation, volume, cancellations, and time from diagnosis to treatment[20]. Other studies have reported reduced duration of stay after surgery[11–13,15,21,22]. A study of 3 hospitals in Norway suggested ringfencing can improve hospital efficiency[14]. A study in Canada found that continuity of care and patient satisfaction could be maintained when introducing additional ring-fenced capacity at a single site[10]."*

    See also response to Reviewer 1 2d.

    b. **This paper initially seems to be suffering from a dearth of academic references. This may seem like a harsh major comment to give, but it is symptomatic of the lack of good academic style in general. Yes, there are papers listed in the bibliography, but they are often not referenced inline in the main body of the article. A lot of work would be required to rewrite this into an academic article, away from what is currently more of a white paper/policy brief.**

Apologies for the hanging references. We have now checked that all listed references are referenced in-line and include DOI where applicable. However, there is a dearth of academic literature around this topic generally, particularly around system-wide effects of ringfencing elective care (most studies are case studies of individual sites). International models are often difficult to compare against as well, as healthcare systems are set up very differently than in the UK, where there is a single payor system. We reflect on this in the Discussion:

> Discussion paragraph 6: "*However, results from these studies are typically not generalisable due to focus on a single site or specialty, or use of weak methods such as before-and-after designs. Moreover, the national healthcare system, local contexts, and even hospital-specific contexts of these studies differs greatly, and it is difficult to extrapolate their findings to English hospital settings."*

c. **Why generalised synthetic controls? Why are they appropriate here? Perhaps most importantly; what are the treated units?! What is the estimand of interest? A lot of key information about this research design is lacking prominence/careful delivery (and accurate technical exposition). When parts of this important information does arrive in the Discussion, it is unfortunately a little too late, and a little too superficial.**

More information on the rationale for choosing the generalised synthetic control method has been added to the Introduction and Methods:

> Introduction paragraph 7: *"The GSC approach is suitable for evaluating the impact of a health care intervention (in this case having a hub) against a counterfactual (if the hub did not exist) where there are multiple treated units, staggered intervention start dates and data is available at multiple time points. This modelling framework effectively accounts for unique aspects of typical health care data, namely, the likely presence of unmeasured confounders and of effects that vary across time and trusts[24,25]."*
>
> Methods, Statistical Analysis*: "GSC offers flexibility for causal inference in our context for the following reasons:*
>
> - *It accounts for unique aspects of typical health care data, namely, the likely presence of unmeasured confounders and of effects that vary across time and trusts. For example, in our data this might include differences between trusts in relation to why they choose to open a hub or not, or other time-varying differences between trusts that could affect outcomes.*
> - *It allows for multiple treated units, enhancing the power to detect effects and allowing mitigating the impact of any potential idiosyncrasies that a few hub trusts may show compared to others.*
> - *It supports varying treatment periods among treated units, which is useful for incorporating staggered hub opening dates in the new-hub trusts analysis.*

- *It conveniently incorporates observed time-varying covariates by including them as main effects in the model.*
- *It provides coherent measures of accuracy, such as standard errors and confidence intervals for effect estimates, using a parametric bootstrap procedure."*

More information on the rationale for choosing the generalised synthetic control method, as well as technical details of the method, are also now included in the Supplementary Material (Appendix 3). See also response to Reviewer 1 3a.

Among aspects we have clarified are the treated units (i.e. hub-trusts) and the target estimand (that is the ATT, or Average Treatment effect on the Treated). To keep the illustration of the generalised synthetic control approach – still a rather sophisticated and nuanced modelling framework – in the main article body accessible we relegated the description of its more technical aspects to the Supplementary Materials.

d. **Unfortunately, a major geographical component is missing from this analysis. Not only is the NHS an increasingly and somewhat intensely regional institution, the regional variations have enormous affect on the demand for services which the NHS supplies, not least the type of services supplied as discussed in this paper. When this does happen -- discussion in terms of elective activity rate -- this is again unfortunately far too light.**

We agree that NHS services are very much a product of regional variation. We have now included a reference to an earlier study that mapped the location and characteristics of hubs across England, including comparing trusts with hubs with trusts nationally (Introduction paragraph 3). That research found trusts with hubs broadly comparable to trusts in England overall, in part because there is wide variation across trusts both with and without hubs.

We also note that the method is robust to national changes in policy or regional variation in trends in the Discussion (paragraph 7).

> Discussion paragraph 7: "*The generalised synthetic control method offers advantages over other causal inference techniques. Most importantly, this methodology does not rely on the parallel trends assumption and can control for time-varying effects, including national changes in policy or regional variation in trends over the study period reducing potential biases in our estimates [23]. Further, by allowing for analysis of multiple trusts simultaneously, we were able to detect effects not apparent in individual trusts. Finally, by deriving the synthetic control empirically, it potentially minimizes researcher bias that can occur with manual selection."*

However, the aim of this work was to understand what the impact of surgical hubs has been across the national healthcare system, and we emphasise this in our discussion by referring to national estimates. We did examine individual variation in effects across trusts (Supplementary Figures 9&10), but largely many of these estimates were not significant or had poor model-fit.

e. **Perhaps most unfortunately, there is absolutely no way a willing replicator of the work could even begin to try to reproduce these results mentioned within; far too much detail is lacking, and details are covered with insufficient depth.**

   Thank you for this comment. The Introduction and Methods sections have now been extensively rewritten. We hope our approach is now clearer and replicable. We have also included much more information on the generalised synthetic control method in the Supplementary Materials Appendix 3. Additionally, we have also published our code on GitHub, which is available here https://github.com/HFAnalyticsLab/elective_hubs.

2. **Minor**

   a. **There are _a lot_ of keywords! Is this in keeping with the journal's guide for authors?**

      We have removed keywords after looking at the guidelines and previous published papers but will defer to the editor as to whether any are needed.

   b. **The first paragraph of the results section would likely better live in the 'Data and Methods' section, where it could be further bolstered. Supplementary Figure 1 goes someway towards this, but a lot more visualisation could be done here to help the reader better understand the dataset(s) and the properties of the estimator's inputs.**

      We have now moved the first few sentences of the Results to the Methods. The Methods has also been carefully revised, which we hope will help with understanding.

   c. **The figures generally leave a little to be desired, both in their production and in their resolution; more work can be done to improve their usefulness, and they can be expanded substantially as expressed above.**

      We have now updated figures to ensure resolution quality is better, and again will defer to the editorial / publishing team to confirm the individual files meet journal requirements. We have also updated axes titles and redesigned some plots for better clarity. Lastly, we have included additional comments in the figure captions to better describe graphed content.

3. **Remarks on code availability**

a. **Not only are there no replication materials available, as mentioned in my review, it would be almost infinitely impossible to even begin to attempt to replicate this study.**

   We have code now publicly available on GitHub, which can be accessed here: https://github.com/HFAnalyticsLab/elective-hubs

Reviewer #1 (Remarks to the Author):

1. **The authors have carefully addressed all my comments and implemented many of my suggestions. I think the paper is now in publishable shape.**

Thank you

Reviewer #2 (Remarks to the Author):

Major comments:

1. **I was glad to receive the chance to again review what is clearly a much improved version of this paper. It certainly addresses some of the earlier points raised but, however, it leaves others relatively insufficiently complete. Primarily, I feel like the referencing is now much more in-line with what would be expected with a journal like Nature Communications. While the subject and writing style matter may or may not be more like a white-paper or specifically that more like one expected from a leading Public Health journal, it is much improved. I commend the authors for this. It feels much more like an academic article than it did previously. Specifically, it much better referenced, and the broader problem statement is more adequately introduced and defined in the opening paragraphs (and it better motivates the need for the study).It also addresses a previous concern about the clear definition of treated and control units.**

Thank you. We address your remaining concerns below

2. **However, a major concern remains regarding the enunciation of the methods. There is almost certainly no way -- as the paper is currently written -- to understand what the GSC method is actually doing. I am of the general opinion that in applied papers, there is no need to formally re-derive a method., or even advance it. However, in this case, the technical detail on exactly what it is doing is so lacking that it makes it hard for a reader outside of the (Generalised) Synthetic Control literature to actually understand what this method does. There are a large number of questions, and equations are necessary to further elaborate things such as 'How are latent factors and loadings generated?', 'How are counterfactual outcomes predicted?', 'How are treatment effects calculated?'. These are not mathematically intractable things to express, and I feel like if they come _after_ the discussion and conclusion of the papers as is common in journals such as this, they would be 'optional things for the reader to consider if and as appropriate' and provide great value. That is to say, again: please consider bringing Appendix 3 into the main text!**

We do not see this as a methods paper and the technical details of the generalised synthetic control method are extensively covered elsewhere in a variety of simulation and impact

evaluation studies, as well as a very informative tutorial by the original author of the method. We have updated the methods in the main manuscript to link explicitly to these resources. We believe that the discussion of the technical aspects of the method is better suited to the Supplementary Material. However, we are happy to discuss this matter further if the editor disagrees. Here is the updated text in the Statistical Analysis sub-section of the Methods section:

Technical details of the GSC method are described extensively in the seminal work by Xu [23] and associated tutorial (https://yiqingxu.org/packages/gsynth/articles/tutorial.html) as well as in simulation and evaluation studies [25,32,34,35]. We also provide more information for the interested reader in Appendix 3 of the Supplementary Material.

3. **I would also raise the fact that HES data represents one specific type of (secondary) care; more should be done to assuage readers about the suitability of this data for this problem. There are, for example, many reasons why HES is not more routinely used in academic research (which is an altogether separate shame), in that it forms just one piece of the (healthcare demand) side of the puzzle (i.e. it omits primary care).**

HES is the main source of data on admissions at NHS Hospitals in England. It includes detailed records of inpatient admissions, and outpatient, and A&E attendances across NHS hospitals in England. This comprehensive coverage allows for robust analysis of hospital-based care which was the focus of this study. Given the evaluation question, this data is suitable for this problem. While HES data may not include primary care information, it can be effectively linked to other routinely available data such as trust population characteristics, as was done in this study.

We have added the following sentence to the Data Sources sub-section of the Methods section for readers unfamiliar with the HES data:

HES is the main source of data on admissions at NHS Hospitals in England. It includes detailed records of all inpatient admissions across NHS hospitals in England.

4. **Further points were raised by both reviewers on the quality of the figures. The new 'Figure 1' is relatively useful, but can likely be much more adequately designed to inform the reader of the 'flow' of the data within the NHS and, accordingly, through the paper (and GSC algorithm). Figma is the relevant tool of choice at this point in time.**

We are pleased Figure 1 is useful. The aim of this logic model is to represent the inputs, activities, outputs and outcomes of the national elective hubs programme to contextualise to the reader the aspect of the programme our evaluation is focused on, and to highlight that there are additional aims which we are unable to evaluate here and are researching in other soon-to-be available work. Flow of data is not appropriate to discuss in this logic model, as it is not a logic model of our evaluation but of the national programme. Elsewhere in the methods, we have justified our decisions to use trust-wide elective surgical procedures as sourced from HES and to construct a generalised synthetic control.

5. **Minor comments: "12 national" --> "twelve national" and "3 hospitals" --> "three hospitals", please! Integers less than twenty one should be words, and integers above 21 should be in numbers (at least outside of the reporting of**

estimated effects). It may be that the editorial team is happy to take care of things like this after the fact.

Thank you for noting this. We have changed the ‘3’ to ‘three’. Common practice is spelling out numbers less than or equal to 10, numerals after that. Can the editor advise?

6. **While the second through figures are useful, they -- like Figure 1 -- should also be lossless (.pdf, .svg) as appropriate.**

All figures are available as pdfs for final submission; we have updated these as per editorial guidelines.

7. **Reviewer #2 (Remarks on code availability): The code looks relatively complete, well written, and well documented. However, it is not a complete replication package due to constraints in data availability (by license).**

Thank you. We have also included (as per editorial guidelines) information in the data availability statement around why this is the case as well.
